# Raltitrexed enhanced antitumor effect of anlotinib in human esophageal squamous carcinoma cells on proliferation, invasiveness, and apoptosis

**DOI:** 10.1186/s12885-023-10691-y

**Published:** 2023-03-04

**Authors:** Hongchao Zhen, Jizheng Tian, Guangxin Li, Pengfei Zhao, Ying Zhang, Juanjuan Che, Bangwei Cao

**Affiliations:** 1 Department of Oncology, Beijing Friendship Hospital, Capital Medical University, #95 Yong An Road, Xicheng District, Beijing, 100050 China; 2 Department of Oncology, Beijing Shunyi District Hospital, Shunyi Teaching Hospital of Capital Medical University, Beijing, 101300 China; 3 Radiation Oncology, Beijing Tsinghua Changgung Hospital, School of Clinical Medicine, Tsinghua University, Beijing, 102218 China; 4 Department of Radiotherapy, Beijing Friendship Hospital, Capital Medical University, Beijing, 100050 China

**Keywords:** Raltitrexed, Anlotinib, Antitumor, ESCC, P-Akt, P-Erk

## Abstract

**Background:**

Anlotinib is a multi-targeted receptor tyrosine kinase inhibitor (TKI) which has exhibited encouraging clinical activity in advanced non-small cell lung cancer (NSCLC) and soft tissue sarcoma. Raltitrexed is well known to be effective in the treatment of colorectal cancer in China. The present study aims to investigate the combinatory antitumor effect of anlotinib and raltitrexed on human esophageal squamous carcinoma cells and further explore the molecular mechanisms in vitro.

**Methods:**

Human esophageal squamous cell lines KYSE-30 and TE-1 were treated with anlotinib or raltitrexed, or both, then cell proliferation was measured by MTS and colony formation assay; cell migration and invasion were detected by wound-healing and transwell assays; cell apoptosis rate was studied by flow cytometry and the transcription of apoptosis-associated proteins were monitored by quantitative polymerase chain reaction (qPCR) analysis. Finally, western blot was performed to check phosphorylation of apoptotic proteins after treatment.

**Results:**

Treatment with raltitrexed and anlotinib showed enhanced inhibitory effects on cell proliferation, migration and invasiveness compared with raltitrexed or anlotinib monotherapy. Meanwhile, raltitrexed combined with anlotinib strongly increased cell apoptosis percentage. Moreover, the combined treatment down-regulated mRNA level of the anti-apoptotic protein Bcl-2 and invasiveness-associated protein matrix metalloproteinases-9 (MMP-9), while up-regulated pro-apoptotic Bax and caspase-3 transcription. Western blotting showed that the combination of raltitrexed and anlotinib could inhibit the expression of phosphorylated Akt (p-Akt), Erk (p-Erk) and MMP-9.

**Conclusions:**

This study indicated that raltitrexed enhanced the antitumor effects of anlotinib on human ESCC cells by down-regulating phosphorylation of Akt and Erk, providing a novel treatment option for patients with esophageal squamous cell carcinoma (ESCC).

**Supplementary Information:**

The online version contains supplementary material available at 10.1186/s12885-023-10691-y.

## Background

Esophageal cancer has been identified as the eighth most common cause of cancer-related death all over the world [1]. Esophageal squamous cell carcinoma (ESCC) accounts for more than 95% of the pathological types of esophageal cancer in China [2]. Despite radical surgery, systemic chemotherapy, and radiotherapy are often administered in clinical practice, disease recurrence often develops within a few years, the five-year survival rate is only 8 to 20% [3, 4]. Since more than one third of patients with esophageal cancer are already advanced at the time of diagnosis, and the overall physical tolerance of patients is slightly poor, it is urgent to develop targeted therapy with less toxic and side effects.

Anlotinib is a novel oral multi-targeted receptor tyrosine kinase inhibitor (TKI), which can effectively inhibit VEGFR, PDGFR, FGFR, C-KIT and other kinases, and has anti-tumor angiogenesis and anti-tumor growth effects [5–8]. Results from a number of previous phase II and III clinical trials have shown that amlotinib exhibits good antitumor activity in a variety of solid tumors, including non-small-cell lung cancer, renal carcinoma, gastric cancer, hepatocarcinoma and soft tissue sarcoma [9]. In addition, anlotinib has been approved by CFDA in 2018 for the treatment of locally advanced or metastatic NSCLC patients who have progressed or relapsed following prior treatment with two systemic chemotherapy treatments. However, there is little experience in the treatment of ESCC patients with anlotinib.

Raltitrexed, also named ZD1694, has been considered as an antimetabolic folate analogue that specifically inhibits thymidylate synthase (TS). TS is a key enzyme in the synthesis of thymidine deoxynucleoside triphosphate (TTP), which is an essential nucleotide for DNA synthesis. Inhibition of TS can lead to DNA breakage and apoptosis [10]. The efficacy of raltitrexed is similar to that of 5-FU, but it can prolong survival and improve quality of life. At present, it is mainly used for the treatment of colorectal cancer patients. In addition, previous studies have demonstrated encouraging antitumor effects of raltitrexed in a variety of tumors, including liver cancer, gastric cancer, malignant mesothelioma, and head and neck cancer, based on its antiproliferative and antiapoptotic activity [11–14].

This is the first attempt to investigate the combined therapeutic effect of raltitrexed and anlotinib on human ESCC and its possible internal mechanism. Since vascular endothelial growth factor receptor-2 (VEGFR-2) is considered to be the primary performer of VEGF-induced antitumor angiogenesis, anlotinib has shown a highly selective inhibitory effect on VEGFR-2 [15–17]. We further hypothesis that the combinatory antitumor effects of raltitrexed and anlotinib are achieved through inhibition of the downstream signaling pathways of VEGFR-2. Two signaling pathways PI3K/Akt/mTOR and Raf/Mek/Erk1/2 are explored as possible mechanisms. Our work will provide a reliable scientific theoretical basis for the combined application of the two drugs in the treatment of human ESCC.

## Methods

### Cell culture and reagents

Human esophageal cancer cell lines KYSE-30 and TE-1 were acquired from the department of experimental research center in Beijing Friendship Hospital (Beijing, China). These two human esophageal squamous cell carcinoma cells were cultured in Roswell Park Memorial Institute (RPMI)-1640 (Hyclone, Termo Scientifc, MA) supplemented with 10% FBS (fetal bovine serum; Biological Industries, Tel Aviv, Israel) and 1% penicillin/streptomycin (KeyGen, Nanjing, China) at 37 °C in a humidified atmosphere of 5% CO_2_. Anlotinib and raltitrexed were kindly given as gifts by Chia Tai Tianqing Co., Ltd. (Nanjing, China). Anlotinib and raltitrexed were dissolved in dimethyl sulfoxide (DMSO, Sigma, St. Louis, USA) and stored at − 20 °C for in vitro studies, and diluted with medium before each test.

### Cell viability assay

The in vitro cytotoxicity were measured by MTS (Vicmed, Xuzhou, Jiangsu, China) assay [18]. Briefly, cells were seeded in 96-well plates at a density of 4 × 10^3^ cells/well at 37 °C overnight. Subsequently, treated with anlotinib or raltitrexed at indicated concentrations or time. After incubating for 24 or 48 h, the cells were washed twice with PBS and then MTS working solution was added into each well and incubated for 1 h at 37 °C according to the manufacturer’s protocol. The resulting absorbance (A) was measured at 490 nm by an microplate reader (Bio-Tek Instruments, USA). The proliferation inhibition rate was calculated by the following formula [cell proliferation inhibition rate (%) = 1-(A value of experimental group-A value of blank group)/(A value of control group-A value of blank group) × 100%]. Triplicate experiments were performed in parallel manner for each concentration point.

### Colony formation assay

ESCC cell lines KYSE-30 and TE-1 were placed into six-well plates at a density of 500 cells/well and treated with appropriate drug conditions (20 μM anlotinib, 2.5 μM raltitrexed, 20 μM anlotinib plus 2.5 μM raltitrexed or its corresponding control) at 37 °C, 5% CO_2_ for approximately 14 days, the medium needs to be replaced every 3–4 days during this period. After 14 days, cells were washed with 1 × PBS, and fixed with 4% paraformaldehyde reagent in methanol for 15 min at room temperature. When visible colonies were observed, the cells were stained with 0.1% crystal violet for 30 min. The colonies were photographed with a digital camera (Olympus, Corporation, Tokyo, Japan) and colonies containing more than 50 cells were counted using the Image J software and the survival fractions were calculated. We took three pictures of each well in this experiment. The percentage rate of colony formation = number of colonies/number of seeded cells × 100%.

### Wound healing assay

Cell migration was assessed by wound healing assay. Cells were seeded into 6-well plates at a density of 6 × 10^5^ cells/well and grown until they reached full confluence. A linear wound was scratched across the cell monolayer with a 200 μL pipette tip. The plates were washed with PBS to remove detached cells, and then fresh medium containing different agents (20 μM anlotinib, 2.5 μM raltitrexed, 20 μM anlotinib plus 2.5 μM raltitrexed or DMSO) were subsequently added to the wells. Cell migration was observed by a light microscope (Olympus, Lake Success, NY, USA) at 0 h, 24 h or 48 h. Migration distance were analyzed using ImageJ software (National Institutes of Health, MD) and three randomly chosen fields were analyzed for each well.

### Transwell invasion assay

Cell invasion assay was performed using transwell coculture chambers inserts with Matrigel (Corning, USA). KYSE-30 and TE-1 cells were re-suspended in the upper chambers at a density of 1 × 10^5^ cells/well and treated with different agents (20 μM anlotinib, 2.5 μM raltitrexed, 20 μM anlotinib plus 2.5 μM raltitrexed or DMSO). About 600 μL of RPMI-1640 per well with 10% fetal bovine serumwas added to the lower chambers. After incubated for 24 h at 37 °C, the invaded cells which passed through the membrane were fixed with 4% paraformaldehyde for 30 min and stained with 0.1% crystal violet (Macklin, Shanghai, China) for 10 min at room temperature. Images were acquired by an inverted microscope (Olympus), and the invaded cells were counted after air-drying in 6 random visual fields for each chamber manually. The average invaded cell numbers per view were calculated, and three independent experiments were carried out in triplicate.

### Cell apoptotic analysis

For apoptosis analysis, KYSE-30 and TE-1 cells were seeded in 6-well plates at a concentration of 1 × 10^6^ cells/well with different agents for 48 h and divided into the four groups: control, anlotinib (20 μM), raltitrexed (2.5 μM) and anlotinib combined with raltitrexed (20 μM + 2.5 μM), the negative control for apoptosis analysis was the equivalent volume of DMSO. Apoptotic cells were distinguished by Annexin V-FITC/7AAD dual staining using apoptosis detection kit (BD Biosciences, Franklin Lakes, NJ, USA). Cells were collected and washed twice with cold PBS and centrifuged for 10 min at 20,000 rpm at 4 °C. Cells were cultured in 300 μL / 1 × 10^6^ cells of 1 × annexin-binding buffer at room temperature for 5 min to prepare the cell samples for flow cytometry, and then double stained with annexin V-FITC (green) and 7AAD-PerCP (red) for 15 min. The percentage of apoptotic cells was analyzed with a FACS Calibur flow cytometer (BD Biosciences) using Flow Jo software (version 9.8.1). The apoptosis rate in every group was described by Q2 (late apoptosis) + Q3 (early apoptosis). All experiments were repeated for three times.

### Quantitative polymerase chain reaction (qPCR) analysis

The qPCR experiments were performed as described previously [19, 20]. The total RNA (Ribonucleic Acid) of cells were extracted using the Trizol buffer (Beyotime) and were reverse-transcribed by Multiscribe™ Reverse Transcriptase (Applied Biosystems, Thermo Scientific Corporation) according to the manufacturer’s instructions. The relative expression was calculated by the comparative Ct method. The primers used for qPCR analysis were shown in Table 1.

**Table 1:** The primers used in this work

Targets	Primer sequence (5′–3′)
**MMP-9**	Forward: 5′-GGGACGCAGACATCGTCATC-3′
	Reverse: 5′-TCGTCATCGTCGAAATGGGC-3′
**Bax**	Forward: 5′-TGAAGCGACTGATGTCCCTG-3′
	Reverse: 5′-CAAAGATGGTCACGGTCTGC-3′
**Caspase-3**	Forward: 5′-CCTGGTTCATCCAGTCGCTT-3′
	Reverse: 5′-TCTGTTGCCACCTTTCGGTT-3′
**Bcl-2**	Forward: 5′-GTGAAGTCAACATGCCTGCC-3′
	Reverse: 5′-ACAGCCTGCAGCTTTGTTTC-3′

### Western blot analysis

KYSE-30 and TE-1 cells were treated with reagents at indicated concentrations and harvested 24 h. Cells were collected and lysed with radio immunoprecipitation assay buffer (RIPA) (Beyotime Biotechnology, Jiangsu, China). Protein concentrations were determined using bicinchoninic acid (BCA) protein assay kit (Merck, Darmstadt, Germany). An equal amount of protein (50 μg) for each sample was resolved using 5% or 10% sodium dodecyl sulfate-polyacrylamide gel electrophoresis (SDS/PAGE) with MOPs running bufer and electrophoretically transferred onto a polyvinylidene difuoride (PVDF) membranes (IPVH00010, Millipore, Massachusetts, USA). After blocking with 5% bovine serum albumin at 37 °C for 1 h, the membranes were incubated with diluted primary antibodies at 4 °C overnight. After washing with cold PBS, the corresponding secondary antibodies were added and followed by ECL detection. The following primary antibodies were used to detect the proteins: Bax (1:1000, Cell Signaling Technology), Bcl-2 (1:1000, Abclonal, Wuhan, China), MMP-9 (1:100, Santa Cruz Biotechnology, Santa Cruz, CA, USA), ERK and p-ERK (1:1000, Cell Signaling Technology), AKT and p-AKT (1:1000, Beverly, MA, USA), VEGFR-2 and p-VEGFR-2 (1:1000, Abcam, Cambridge, UK). Subsequently, the membranes were incubated with anti-rabbit IgG conjugated to HRP secondary antibody (1:5000; Cell Signaling Technology, Inc.) for 1.5 h at room temperature. Finally, immunoreactive bands after incubation with secondary antibodies conjugated to peroxidase were detected using an ECL kit (Beyotime, Nangjing, Jiangsu, China) according to the manufacturer’s instructions. Bands were imaged by using the Fusion Fx7 imager (Vilber Lourmat, France) and analyzed with the ImageJ software (NIH, Bethesda, MD). We tailored the membranes, the upper and lower boundaries according to protein molecular weight, and the left and right boundaries according to different cell lines.

### Statistical analysis

All statistical analysis was performed using the GraphPad Prism software package version 6.0 (GraphPad Software, Inc., La Jolla, CA, USA). All data were collected from three independent experiments and expressed as the mean ± standard deviation (SD). Differences between groups were analyzed using one-way analysis of variance (ANOVA). The statistical differences were evaluated by SPSS 20.0 soft ware (IBM Corp., Armonk, NY, USA). *P* value less than 0.05 (*P* < 0.05) was considered statistically significant.

## Results

### Anlotinib combined with raltitrexed inhibited cell proliferation

**Fig. 1:**
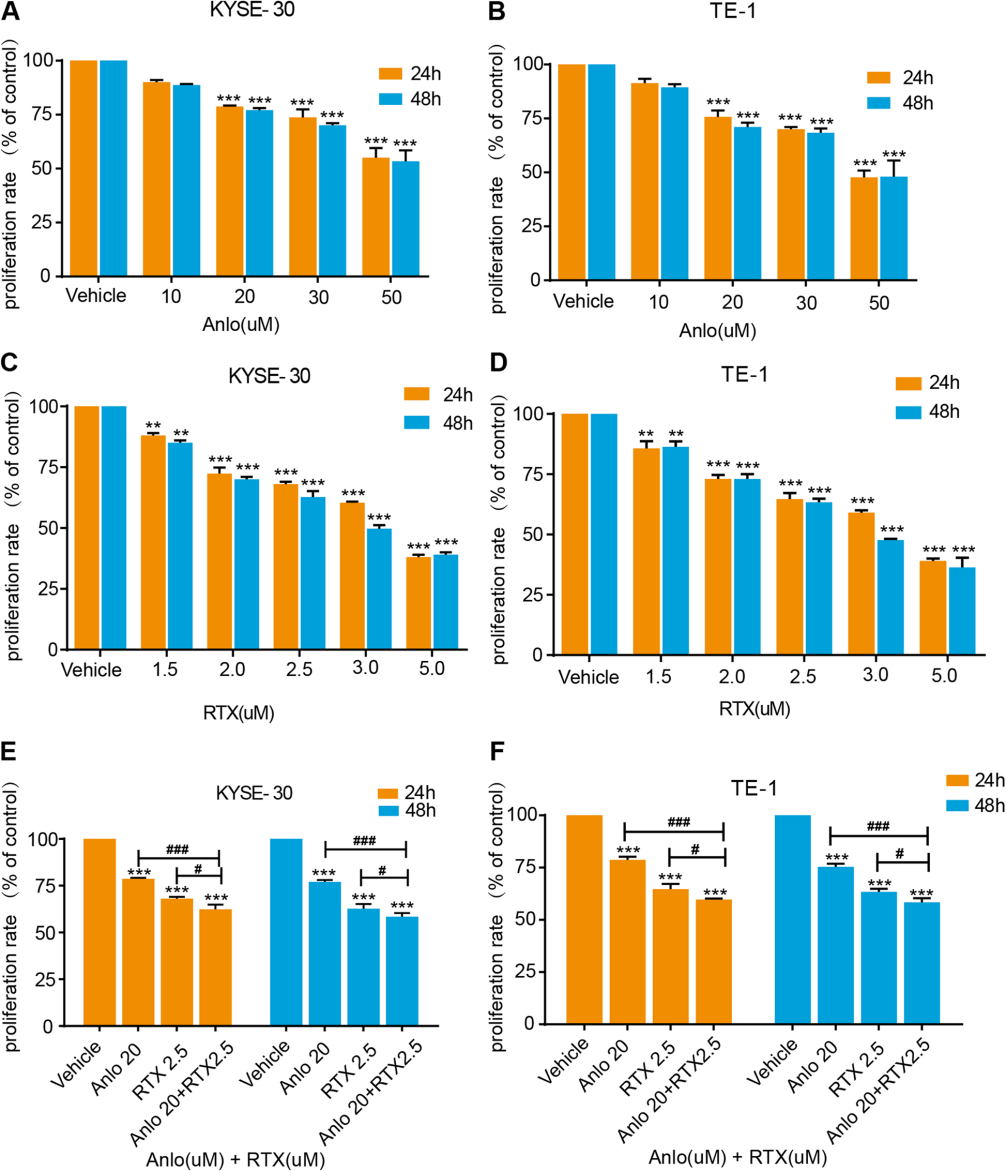
Combinated effects of raltitrexed and anlotinib on cell proliferation in ESCC cells. **A** and **B** Anlotinib reduced cell proliferation rates of KYSE-30 (**A**) and TE-1 (**B**) cells in a dose-dependent manner after 24 h and 48 h treatment. **C** and **D** Raltitrexed reduced cell proliferation rates of KYSE-30 (**C**) and TE-1 (**D**) cells in a dose-dependent manner after 24 h and 48 h treatment. **E** and **F** Cell proliferation rates of KYSE-30 (**E**) and TE-1 (**F**) cells after treated with control, 20 μM anlotinib, 2.5 μM raltitrexed or 20 μM anlotinib + 2.5 μM raltitrexed for the indicated time in MTS assays. Data indicate means + SD of three biological replicates. Student’s t test; ^**^P < 0.01, ^***^P < 0.001 (versus control); ^*#*^*P* < 0.05, ^*###*^*P* < 0.001 (versus 20 μM anlotinib or 2.5 μM raltitrexed)

At present, the treatment of tumors is progressing rapidly, but the treatment effect of advanced ESCC is still not ideal and the prognosis is poor. It is worth exploring whether the combination of anlotinib and raltitrexed, a conventional chemotherapy drug, can produce synergistic effects in the treatment of ESCC. To test the efficacy of anlotinib, raltitrexed and the combination of drugs on the proliferation of ESCC cells, we co-cultured different concentrations of anlotinib and raltitrexed with KYSE-30 and TE-1 cell lines which expressed the vascular endothelial growth factor receptor, VEGFR-2 [21–23]. As shown in Fig. 1A and B, anlotinib treatment on KYSE-30 and TE-1 cells at a concentrations of 10 μM, 20 μM, 30 μM and 50 μM decreased the cell proliferation rate after 24 or 48 hours of incubation. And it became significantly different from the control group when incubating with 20 μM anlotinib after 24 or 48 hours of incubation. Similarly, when incubated with raltitrexed (Fig. 1C and D) at concentrations of 1.5 μM, 2.0 μM, 2.5 μM, 3.0 μM and 5.0 μM, a significant reduction effect appeared after incubating 24 or 48 hours in comparison to the control. These data indicate both anlotinib and raltitrexed reduced the viability of ESCC cells in a concentration-dependent manner by MTS assay. Based on the above results as well as previous data from our research group [23–25], we chose 20 μM anlotinib and 2.5 μM raltitrexed to treat KYSE-30 cells and checked if any combinatory effect existed. As is shown in Fig. 1E, results revealed that anlotinib at 20 μM combined with raltitrexed at 2.5 μM produced stronger inhibition on cell proliferation compared with the anlotinib (*P* < 0.001) and raltitrexed (*P* < 0.05) monotherapy groups after incubation with both reagents for 24 h or 48 h. Exactly similar effects were gained for the same treatment on TE-1 cells (Fig. 1F).

**Fig. 2:**
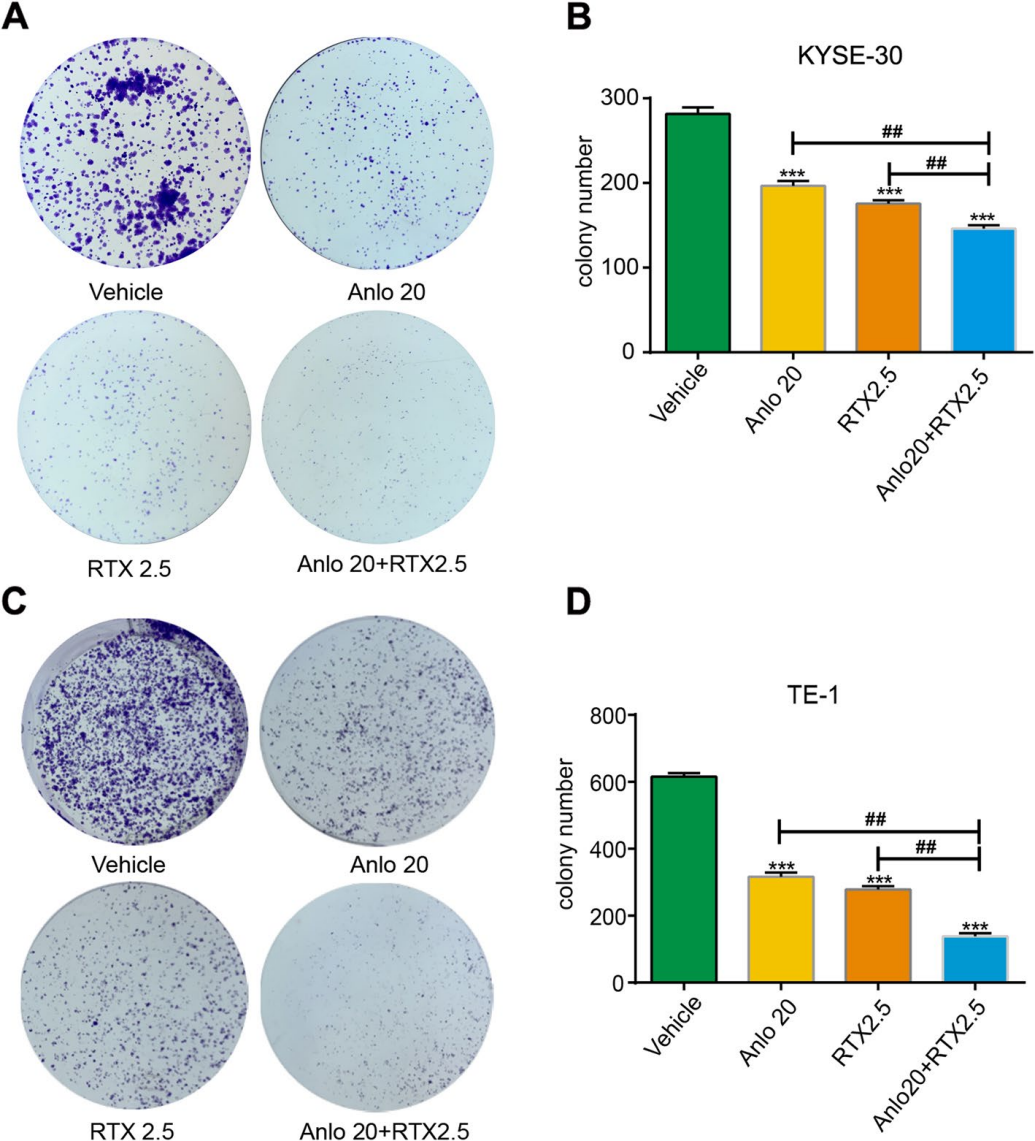
Combinated effects of raltitrexed and anlotinib on viability of ESCC cells. **A** and **C** Colony formation ability of KYSE-30 (**A**) and TE-1 (**C**) cells inhibited by control, 20 μM anlotinib, 2.5 μM raltitrexed or 20 μM anlotinib + 2.5 μM raltitrexed. **B** and **D** Quantitative analysis of the number of colonies in KYSE-30 (**B**) and TE-1 (**D**) cells. Data indicate means + SD of three biological replicates. Student’s t test; ^***^P < 0.001 (versus control); ^##^*P* < 0.01 (versus 20 μM anlotinib or 2.5 μM raltitrexed)

Subsequently, the anti-proliferation effects of anlotinib and raltitrexed on ESCC cells were further verified by colony formation assay. According to the results of the MTS study, 20 μM anlotinib and 2.5 μM raltitrexed were selected as the final concentration of our study, and each cell line was incubated for 14 days with anlotinib or raltitrexed monotherapy or the combination group. From the results in Fig. 2, we observed that the number of colonies formed in the anlotinib and raltitrexed monotherapy groups were significantly decreased than the control group (*P* < 0.001), while the number of colonies formed in the combination group became almost invisible in the combination group compared with the anlotinib and raltitrexed monotherapy groups (all *P* < 0.01) in KYSE-30 (Fig. 2A and B) and TE-1 (Fig. 2C and D) cells. Combined with the above results, we concluded that raltitrexed enhanced the anti-proliferation ability of anlotinib on ESCC cells.

### Anlotinib combined with raltitrexed enhanced the inhibition of cell migration and invasion

**Fig. 3:**
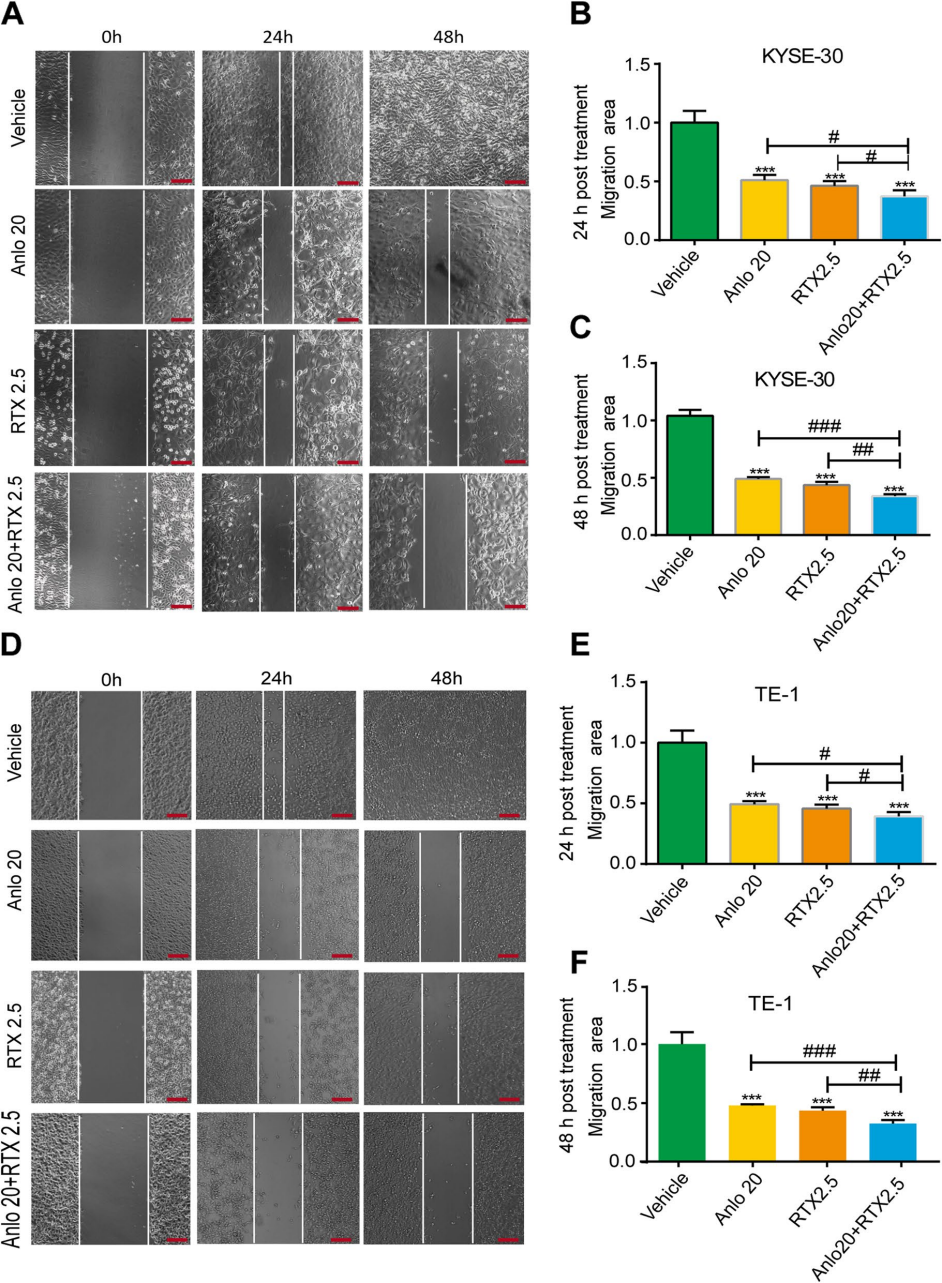
Combinated effects of raltitrexed and anlotinib on cell migration in ESCC cells. **A** and **D** KYSE-30 (**A**) and TE-1 (**D**) cells treated with control, 20 μM anlotinib, 2.5 μM raltitrexed or 20 μM anlotinib + 2.5 μM raltitrexed were scraped and imaged immediately (0 h) and later (24 h and 48 h), and images of the wound gap were taken. Scale bar = 100 μm. Quantitative analysis of the wound healing rate in KYSE-30 (**B** and **C**) and TE-1 (**E** and **F**) cells. Data indicate means + SD of three biological replicates. Student’s t test; ^***^P < 0.001 (versus control); ^*#*^*P* < 0.05, ^##^*P* < 0.01, ^###^*P* < 0.001 (versus 20 μM anlotinib or 2.5 μM raltitrexed)

The migration and invasion ability of tumor cells are closely related to the occurrence of distant metastasis. We used wound-healing and transwell assays to evaluate the effects of 20 μM anlotinib, 2.5 μM raltitrexed alone or both on migration and invasion of ESCC cells. As shown in Fig. 3, both 20 μM anlotinib and 2.5 μM raltitrexed monotherapy effectively inhibited cell migration in KYSE-30 (Fig. 3A-C) and TE-1 (Fig. 3D-F) cells compared with the control group after scratching for 24 h and 48 h (all *P* < 0.001). Meanwhile, the inhibitory effects were more obvious in the combination group compared with 20 μM anlotinib and 2.5 μM raltitrexed monotherapy groups.

**Fig. 4:**
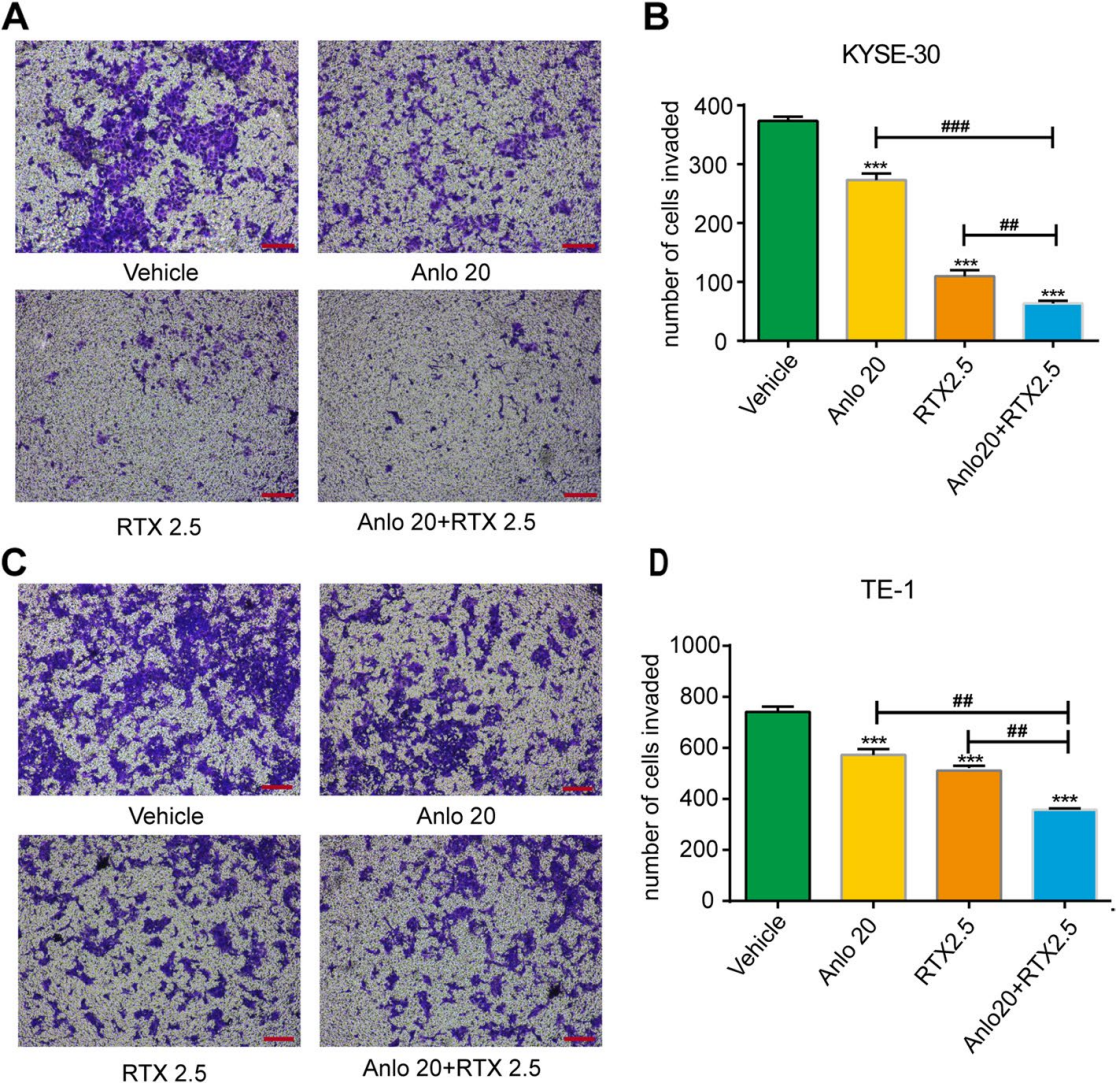
Combinated effects of raltitrexed and anlotinib on cell invasion in ESCC cells. **A** and **C** After treatment with control, 20 μM anlotinib, 2.5 μM raltitrexed or 20 μM anlotinib + 2.5 μM raltitrexed for 24 h, invaded cells were stained and counted using microscopy in KYSE-30 (**A**) and TE-1 (**C**) cells. **B** and **D** Quantitative analysis of the number of invaded cells in KYSE-30 (**B**) and TE-1 (**D**) cells. Data indicate means + SD of three biological replicates. Student’s t test; ^***^P < 0.001 (versus control); ^##^*P* < 0.01, ^###^*P* < 0.001 (versus 20 μM anlotinib or 2.5 μM raltitrexed)

Consistent with the wound-healing results, we also observed anlotinib and raltitrexed monotherapy inhibited cell invasion through transwell assay in KYSE-30 (Fig. 4A and B) and TE-1 (Fig. 4C and D) cells compared with the control group after statistical analysis (all *P* < 0.001). It is worth noting that the inhibitory effect in cell invasion was further reduced by combined treatment of 20 μM anlotinib and 2.5 μM raltitrexed compared with anlotinib and raltitrexed monotherapy. MMP-9 is a major member of the zinc metalloproteinase family because it stimulates cancer metastasis by degrading ECM and collagens, facilitating cancer cell invasion and metastasis [26]. Therefore, MMP-9 is a potentially key molecule in cancer invasion and is considered a target for drug development. In order to explore the mechanisms behind the results above, the level of MMP-9 mRNA was detected by qPCR. As shown in Fig. 6B and F, the level of MMP-9 mRNA expression were significantly decreased following treatment with 20 μM anlotinib and 2.5 μM raltitrexed in KYSE-30 and TE-1 cells (all *P* < 0.01), and when anlotinib was combined with raltitrexed, further inhibition in MMP-9 expression was observed (*P* < 0.01). Similar conclusions were found in the results of western blot, as shown in Fig. 7D and L. After 24 hours of anlotinib and raltitrexed treatment of KYSE-30 and TE-1 cell lines, the expression level of MMP-9 showed a significant downward trend compared with the control group, and the downward trend was more significant in the combination group than in 20 μM anlotinib and 2.5 μM raltitrexed monotherapy group (*P* < 0.01). Taken together, these results demonstrated that raltitrexed enhanced the ability of anlotinib to inhibit the migration and invasion of ESCC cells.

### Anlotinib combined with raltitrexed increased cell apoptosis

**Fig. 5:**
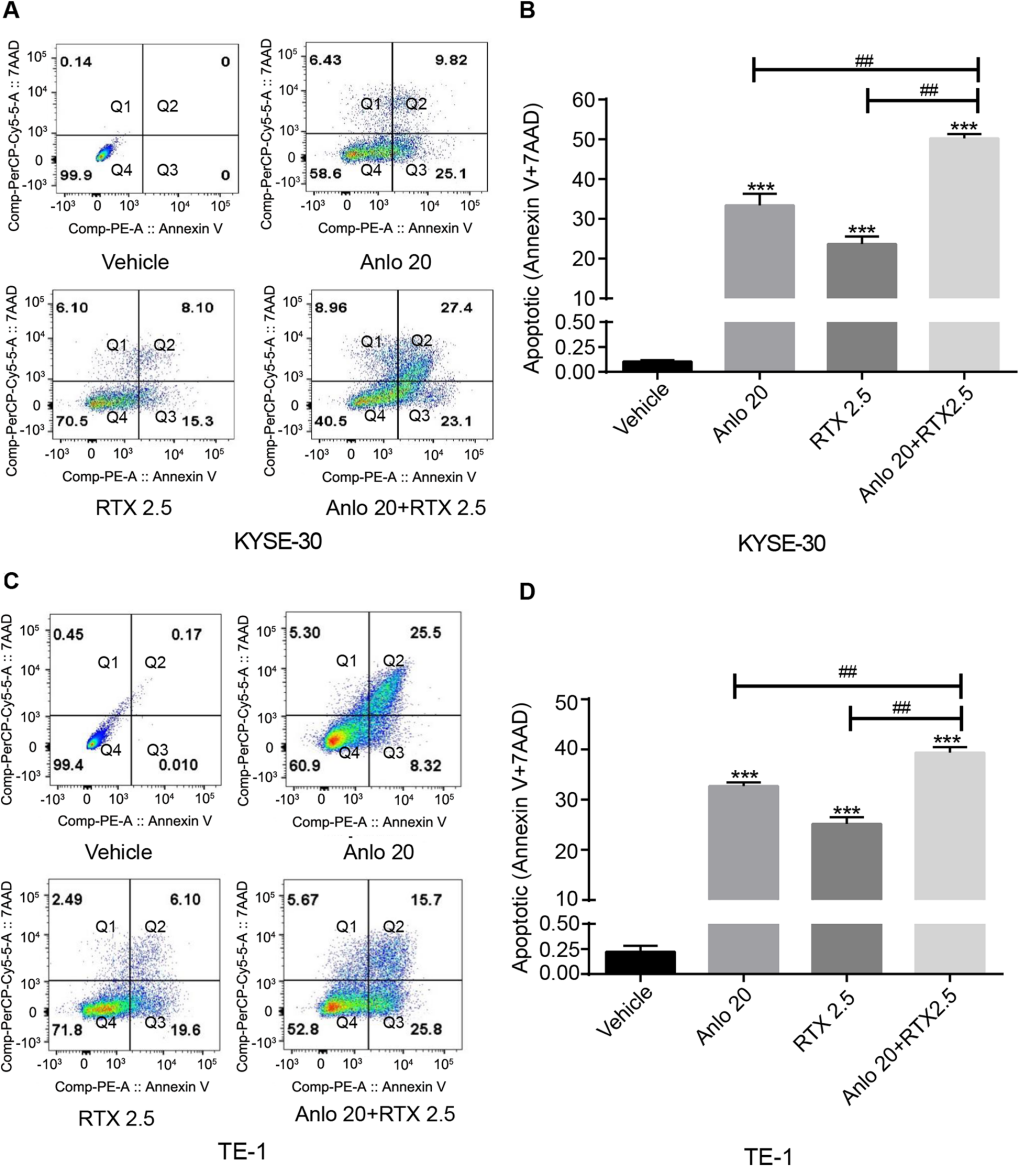
Combinated effects of raltitrexed and anlotinib on cell apoptosis in ESCC cells. **A** and **C** KYSE-30 (**A**) and TE-1 (**C**) cells were exposed to control, 20 μM anlotinib, 2.5 μM raltitrexed or 20 μM anlotinib + 2.5 μM raltitrexed for 48 h before Annexin V-FITC and 7AAD-PerCP staining and apoptosis percentage was detected by flow cytometry. **B** and **D** Quantitative analysis of the apoptotic rate in KYSE-30 (**B**) and TE-1 (**D**) cells. Q1 (necrosis), Q2 (late apoptosis), Q3 (early apoptosis) and Q4 (viable). Data indicate means + SD of three biological replicates. Student’s t test; ^***^P < 0.01 (versus control); ^*##*^*P* < 0.01 (versus 20 μM anlotinib or 2.5 μM raltitrexed)

To reveal the effects of anlotinib and raltitrexed on cell apoptosis in human esophageal squamous cells, Annexin V and 7AAD staining assay were performed to detect apoptosis rates of KYSE-30 and TE-1 cell lines treated with various reagents for 24 h and then the apoptosis percentage was analyzed by flow cytometry. As shown in Fig. 5, the apoptosis rates of KYSE-30 (Fig. 5A and B) and TE-1 (Fig. 5C and D) cells in both anlotinib and raltitrexed monotherapy groups were significantly higher than those in the control group (all *P* < 0.001), indicating both of them had strong apoptosis-inducing effects in ESCC cells. More obviously, the apoptosis rates of the combination treatment group were significantly higher than anlotinib and raltitrexed monotherapy groups (all *P* < 0.01), suggesting that combined treatment could further induce apoptosis.

**Fig. 6:**
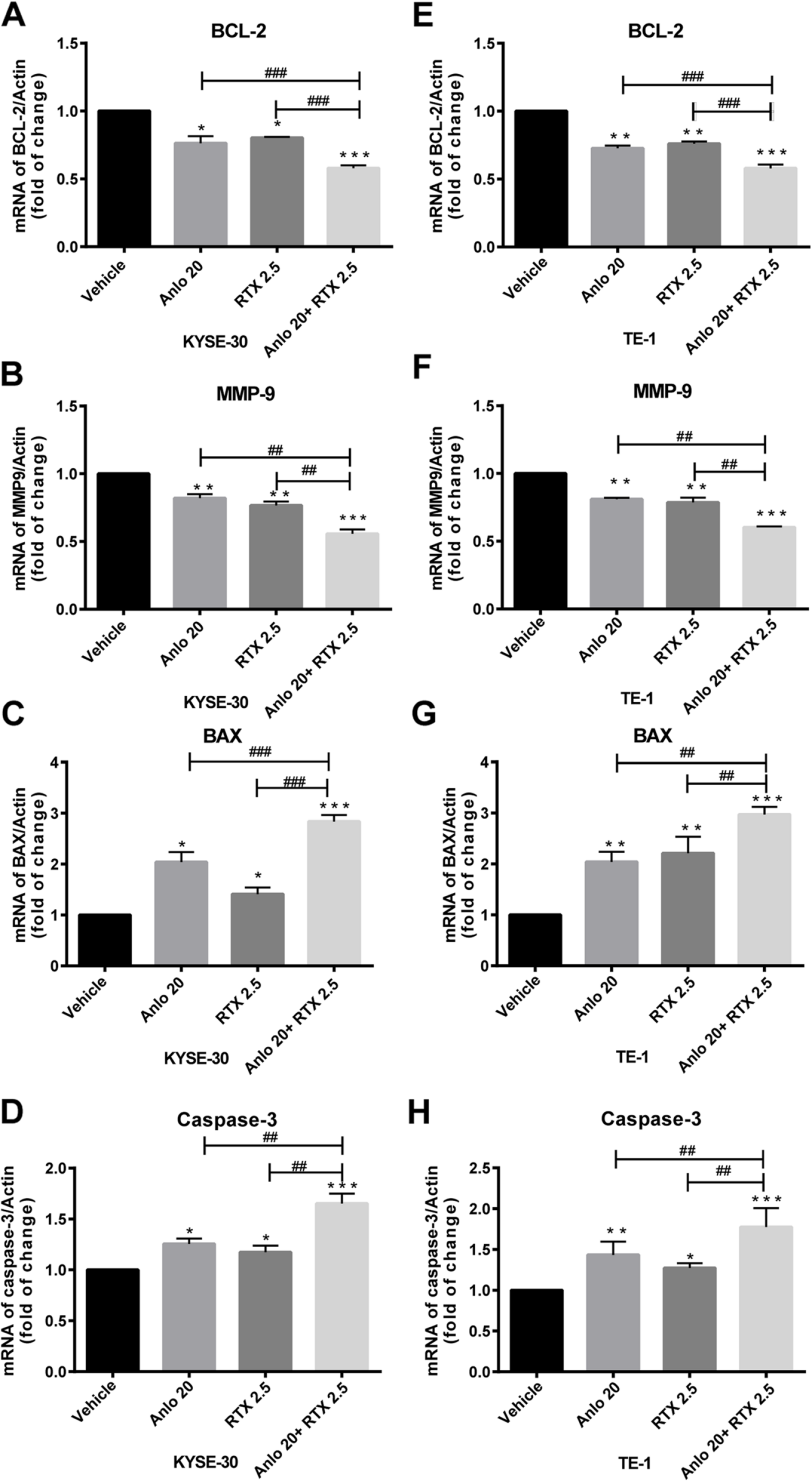
Combinated effect of raltitrexed and anlotinib on gene transcription in ESCC cells. mRNA levels of Bcl-2, MMP-9, Bax and Caspase-3 were measured by qPCR in either KYSE-30 (**A-D**) or TE-1 (**E-H**) cells after treated by 20 μM anlotinib, 2.5 μM raltitrexed, or both. Data indicate means + SD of three biological replicates. Student’s t test; ^*^P < 0.05, ^**^P < 0.01, ^***^P < 0.01 (versus control); ^*##*^*P* < 0.01, ^*###*^*P* < 0.001 (versus 20 μM anlotinib or 2.5 μM raltitrexed)

**Fig. 7:**
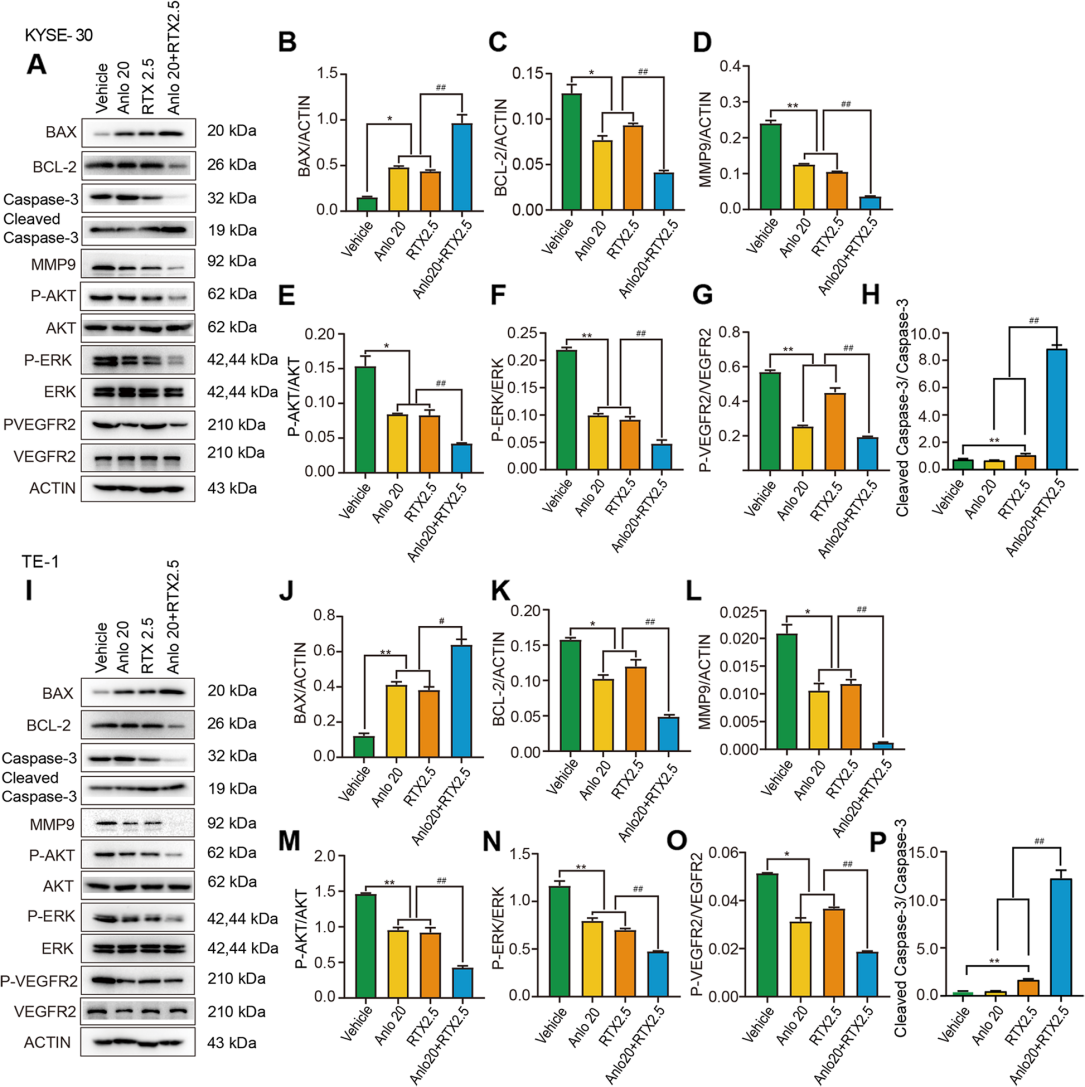
Protein expression after various treatments detected and quantified by Western blot analysis. KYSE-30 (**A-H**) and TE-1 (**I-P**) cells were treated with various treatments for 24 h. The protein levels of BAX, BCL-2, MMP-9, p-AKT, AKT, p-ERK, ERK, p-VEGFR-2, VEGFR-2, Caspase-3 and Cleaved Caspase-3 were determined by western blot analysis. β-ACTIN was detected as loading control. ^*^P < 0.05, ^**^P < 0.01 (versus control); ^*#*^*P* < 0.05, ^*##*^*P* < 0.01 (versus 20 μM anlotinib or 2.5 μM raltitrexed)

Subsequently, we further used qPCR method to investigate the effects of anlotinib and raltitrexed monotherapy and the combination therapy on the transcription levels of apoptosis-related protein in KYSE-30 and TE-1 cell lines. Results as shown in Fig. 6, compared with the control group, the expression levels of pro-apoptotic protein Bax (Fig. 6C and G) and caspase-3 (Fig. 6D and H) mRNA were significantly increased in anlotinib and raltitrexed monotherapy groups, and the elevation became more noticeable in the combination treatment group compared with anlotinib and raltitrexed monotherapy groups. On the contrary, the expression level of anti-apoptotic protein Bcl-2 mRNA decreased in both anlotinib and raltitrexed monotherapy groups compared with the control group, KYSE-30 (*P* < 0.05) (Fig. 6A) and TE-1 (*P* < 0.01) (Fig. 6E), and the decrease was more significant in the combination group than in anlotinib and raltitrexed monotherapy groups (all *P* < 0.001). Consistent with the results assayed by qPCR method, western blot analysis showed the expression of Bax and cleaved caspase-3 were increased in the combination group in both KYSE-30 (Fig. 7B and H) and TE-1 (Fig. 7J and P) cells compared with anlotinib and raltitrexed monotherapy groups. Conversely, the quantitative results in Fig. 7C and K also indicated that the expression of Bcl-2 was decreased in both anlotinib and raltitrexed monotherapy groups compared with the control group (*P* < 0.05), and the down regulation became more significantly in the combination group compared with anlotinib and raltitrexed monotherapy groups (*P* < 0.01). Taken together, these results support that retitrexed enhances the apoptosis induced by anlotinib.

### Anlotinib combined with raltitrexed downregulated the expression of p-Akt and p-Erk

Western blot analysis was carried out to clarify the antitumor molecular mechanisms of anlotinib and raltitrexed. We examined PI3K/Akt and Ras/Erk, two major signaling pathways downstream of VEGF signaling pathway [27]. After 24 hours of treatment, it turned out that the expression of phosphorylated Akt (p-Akt) and Erk (p-Erk), which represent the activity of Akt and Erk signaling pathways, were inhibited noticeably by 20 μM anlotinib and 2.5 μM raltitrexed respectively, and the combinatory treatment further decreased p-Akt (Fig. 7E and M) and p-Erk (Fig. 7F and N) level compared with anlotinib and raltitrexed monotherapy group (all *P* < 0.01). In addition, the level of phosphorylated VEGFR-2 expression was significantly reduced in the anlotinib and raltitrexed monotherapy groups compared with the control group in both KYSE-30 and TE-1 cells, and the decrease was more significant in the combination group compared with the monotherapy group (Fig. 7G and O) (all *P* < 0.01). These results suggest that raltitrexed may enhance the antitumor effect of anlotinib on human ESCC cells by inhibiting Akt and Erk signaling pathways and affecting the phosphorylation of VEGFR-2.

## Discussion

Although in the past decades, the choice of treatment options for advanced ESCC has been greatly developed, getting rid of the constraints of conventional chemotherapy regimens, the overall treatment effect of ESCC patients still needs to be improved due to the characteristics of drug resistance and highly invasive of ESCC, as well as the reduced physical endurance of patients after repeated chemotherapy.

The concept of tumor growth relied on the formation of tumor blood vessels was proposed by professor Folkman in 1971, which suggested that anti-tumor angiogenesis would be a potential strategy for cancer treatment [28]. Vascular endothelial growth factor receptors (VEGFRs) play an important role in pathological angiogenesis, VEGF-targeting has emerged as a viable therapeutic options for several malignancies [29]. VEGFR-2 is functionally activated through autophosphorylation of its carboxyl terminus and subsequently stimulates PI3K/Akt and Ras/Erk signaling pathways mediated cell proliferation and metastasis. Anlotinib is an orally, multi-targeted small molecule TKI that blocks VEGFR-2 phosphorylation. Previous clinical trials have demonstrated antitumor activity of anlotinib in a variety of solid tumors. Nevertheless, there are very few studies detailing the clinical efficacy of anlotinib in patients with ESCC. At the same time, with the extension of treatment time, drug resistance becomes an inevitable clinical problem. In addition, oral TKI alone can not achieve the optimal anti-tumor effect. 5-FU is an indispensable routine drug in chemotherapy for ESCC patients. However, drug side effects have been encountered in numerous patients, therefore, it is necessary to establish alternatives with lower levels of toxicity. Different from 5-FU, raltitrexed is a more suitable choice for ESCC patients with a history of heart disease because of its low incidence of cardiotoxicity and digestive system adverse reactions [30, 31]. In our study, we conducted cytological studies of antitumor effects of anlotinib and raltitrexed in two VEGFR-2 positive ESCC cell lines KYSE-30 and TE-1, and we found that the combination of anlotinib and raltitrexed significantly enhanced antitumor effects compared with anlotinib and raltitrexed monotherapy.

In our study, we confirmed the proliferation inhibition effect of anlotinib and raltitrexed on human ESCC cell lines by MTS and colony formation methods, and we observed that the combination of anlotinib and raltitrexed was more effective than anlotinib and raltitrexed monotherapy groups (Figs. 1 and 2). Therefore, we believe that anlotinib combined with raltitrexed can be used as a treatment option for ESCC patients. In addition, we used wound-healing and transwell experiments to confirm that both anlotinib and raltitrexed could inhibit the migration and invasion of ESCC cells KYSE-30 and TE-1, and the inhibitory effect of the combination therapy was better than anlotinib and raltitrexed monotherapy groups (Figs. 3 and 4). In order to reveal the mechanism, we further detected the expression level of invasion-related protein MMP-9 and its mRNA transcription level by western blot and qPCR methods. The results showed that both of anlotinib and raltitrexed could inhibit the expression of MMP-9 protein and inhibit transcription, and the inhibition effect was stronger in the combination group (Figs. 6 and 7). Based on the above findings, we concluded that anlotinib inhibits the migration and invasion of human ESCC cell lines KYSE-30 and TE-1 by inhibiting the expression of MMP-9, and the addition of raltitrexed enhances the inhibitory effect of anlotinib on cell migration and invasion.

In addition, flow cytometry results showed that compared with anlotinib or raltitrexed monotherapy group, the apoptotic rate of KYSE-30 and TE-1 cells in the combination treatment group was significantly increased (Fig. 5). To further verify the results of flow cytometry, qPCR assay was used to detect the mRNA expression levels of anti-apoptotic protein Bcl-2 and pro-apoptotic proteins Bax and caspase-3. The results showed that the mRNA expression of Bcl-2 were decreased in anlotinib and raltitrexed groups. The reduction were more pronounced in the combination treatment group. On the contrary, the expression levels of Bax and caspase-3 mRNA in anlotinib or raltitrexed monotherapy groups were higher than those in the control group, and were further increased in the combination treatment group (Fig. 6). The western blot analysis showed similar results (Fig. 7). These conclusions indicated that raltitrexed could enhance the promoted apoptosis effect of anlotinib on human ESCC cell lines KYSE-30 and TE-1 by affecting the expression of apoptosis-related proteins.

Previous studies have shown that overactivation of p-Akt and p-Erk could inhibit the apoptosis of tumor cells and promote cell proliferation and invasion. In our study, we found anlotinib and raltitrexed decreased the level of p-Akt and p-Erk expression, and anlotinib combined with raltitrexed inhibited p-Akt and p-Erk expression in even greater extent (Fig. 7). Based on our findings, we believe that raltitrexed potentiates the antitumor effects of anlotinib on KYSE-30 and TE-1 cells by blocking PI3K/Akt and Ras/Erk signaling pathways.

It is well known that the histopathological types of esophageal cancer differ significantly between eastern and western countries. Adenocarcinoma is the main type in western countries, while in China, more than 95% of esophageal cancers are esophageal squamous cell carcinoma (ESCC) [2]. Therefore, due to the different biological behaviors, patients with ESCC in Chinese population should have unique treatment options and choices, which are different from esophageal adenocarcinoma. Cisplatin combined with 5-FU or paclitaxel is the standard first-line treatment for patients with advanced ESCC. However, cisplatin combined with 5-FU regimen only resulted in 5.5 months of progression-free survival (PFS) and 10 months of overall survival (OS) benefit in patients with advanced ESCC [32]. The PFS of cisplatin plus paclitaxel regimen was 6 months, and the OS was extended to 12 months. As second-line treatment options, the median PFS and OS of irinotecan and docetaxel could reached 3 months and 7.1 months, respectively [33]. There is still lack of standard treatment options for third-line and posterior treatment of ESCC. In particular, research data on the use of targeted drugs in advanced ESCC are relatively limited. In the results of the Phase II clinical trial of ALTER1102, we saw promising results with anlotinib as a second-line treatment for advanced ESCC, with an objective response rate (ORR) of 7.34%, median PFS of 3.02 months and OS of 6.11 months, respectively [34]. After the early multi-line chemotherapy, the physical condition of most patients already decline, so, the multi-target oral small molecule TKI maybe a good treatment option.

With the data described above, our study further revealed the inhibitory effect of anlotinib on ESCC cells through in vitro cytological experiments, and confirmed that the combination of raltitrexed and anlotinib could produce a synergistic effect, which provides more treatment options for ESCC posterior line treatment. From an objective point of view, limitations were still observed in our study. Firstly, our conclusions are limited to the cytological level at present. In the future, we will conduct animal experiments and further promote clinical application to verify our conclusions. The ultimate purpose of in vitro and in vivo drug research is to serve clinical treatment, so we need to pay more attention to whether the results of cytology experiments and animal experiments can be applied to clinical treatment. A case report confirmed that a 57-year-old man with recurrent ESCC after surgery, the patient had a failed immunotherapy course, but benefited from anlotinib combined with chemotherapy [nedaplatin (110 mg) and raltitrexed (4 mg) plus anlotinib (12 mg, D1-D14, Q3W)] for a fourth-line therapy. Survival after the combined therapy was greater than 19 months, and the overall patient survival was greater than 32 months [35]. The results confirmed that anlotinib combined with raltitrexed is effective in the posterior line treatment of advanced ESCC patients. Of course, more clinical cases are needed to confirm the conclusion. In addition, whether the incidence of adverse reactions increases after the combination of anlotinib and raltitrexed will be the focus of our follow-up observation. Secondly, with respect to raltitrexed increasing the antitumor efficacy of anlotinib in ESCC cells, besides the mechanism we found above, whether there are other possible underlying mechanisms is still worth further exploration in our future work. Finally, the combinatory efficacy of anlotinib and raltitrexed with other therapy methods, such as immune checkpoint inhibitors for ESCC treatment also need to be studied.

## Conclusion

Our study first demonstrated that anlotinib combined with raltitrexed produced significant inhibition on cell proliferation, migration, and invasion in ESCC cell lines KYSE-30 and TE-1 by down-regulating the expression of p-Akt and p-Erk. Our results will provide an important reference for the application of targeted drugs toward ESCC, and the combination of anlotinib and raltitrexed might be a potential therapeutic mixture in ESCC patients. However, the clinical application of anlotinib and raltitrexed is hampered by the lack of clinical trial evaluation and should be studied in randomized controlled clinical trials in the future.

## Supplementary Information

**Additional file 1.**

## Data Availability

The datasets used and/or analyzed during the current study are available from the corresponding author on reasonable request.

## Abbreviations

ESCC: Esophageal squamous cell carcinoma
TKI: Tyrosine kinase inhibitor
NSCLC: Non-small cell lung cancer
qPCR: Quantitative polymerase chain reaction
MMP-9: Matrix metalloproteinases-9
TS: Thymidylate synthase
TTP: Thymidine deoxynucleoside triphosphate
VEGFR-2: Vascular endothelial growth factor receptor-2
PFS: Progression-free survival
OS: Overall survival
ORR: Objective response rate.
