# Supplementary material for: Raltitrexed enhanced antitumor effect of anlotinib in human esophageal squamous carcinoma cells on proliferation, invasiveness, and apoptosis
Source: BMC Cancer. 2023 Mar 4;23:207. doi: 10.1186/s12885-023-10691-y (PMC9985835; doi:10.1186/s12885-023-10691-y)
Supplement: Supplementary file 1 — Additional file 1. [file 12885_2023_10691_MOESM1_ESM.pdf]

Raltitrexed enhanced antitumor effect of anlotinib in human esophageal squamous carcinoma cells on proliferation, invasiveness, and apoptosis

Hongchao Zhen[1], Jizheng Tian[2], Guangxin Li[3], Pengfei Zhao[4], Ying Zhang[1], Juanjuan Che[1], Bangwei Cao[1*]

BAX

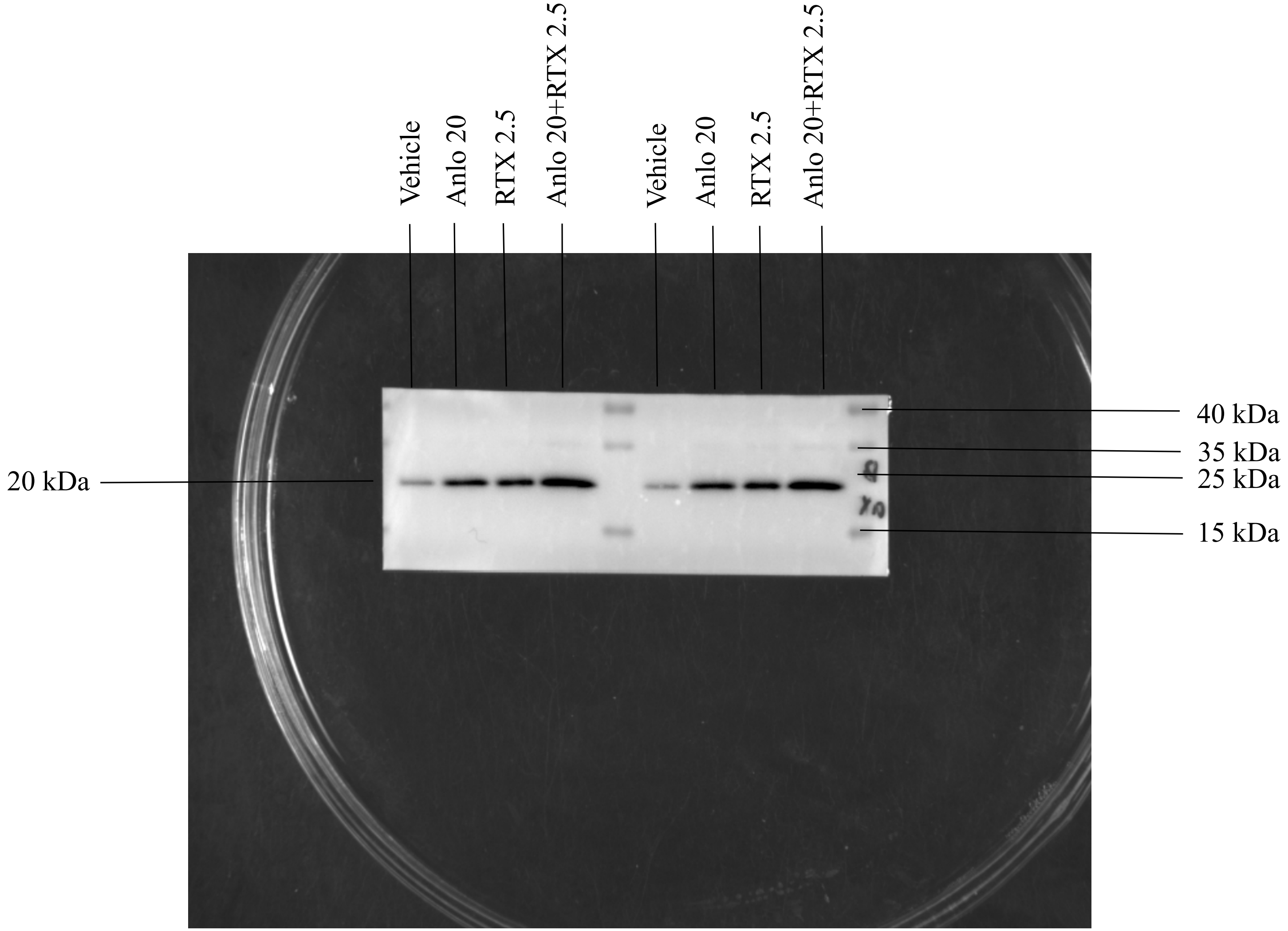

The original Western Blot images of Protein BAX in Figure 7. From left to right: (Left:TE-1) : Vehicle, Anlo 20, RTX 2.5, Anlo 20+RTX 2.5; (Right:KYSE-30): Vehicle, Anlo 20, RTX 2.5, Anlo 20+RTX 2.5. The expected molecular weight is 20 kDa.

BCL-2

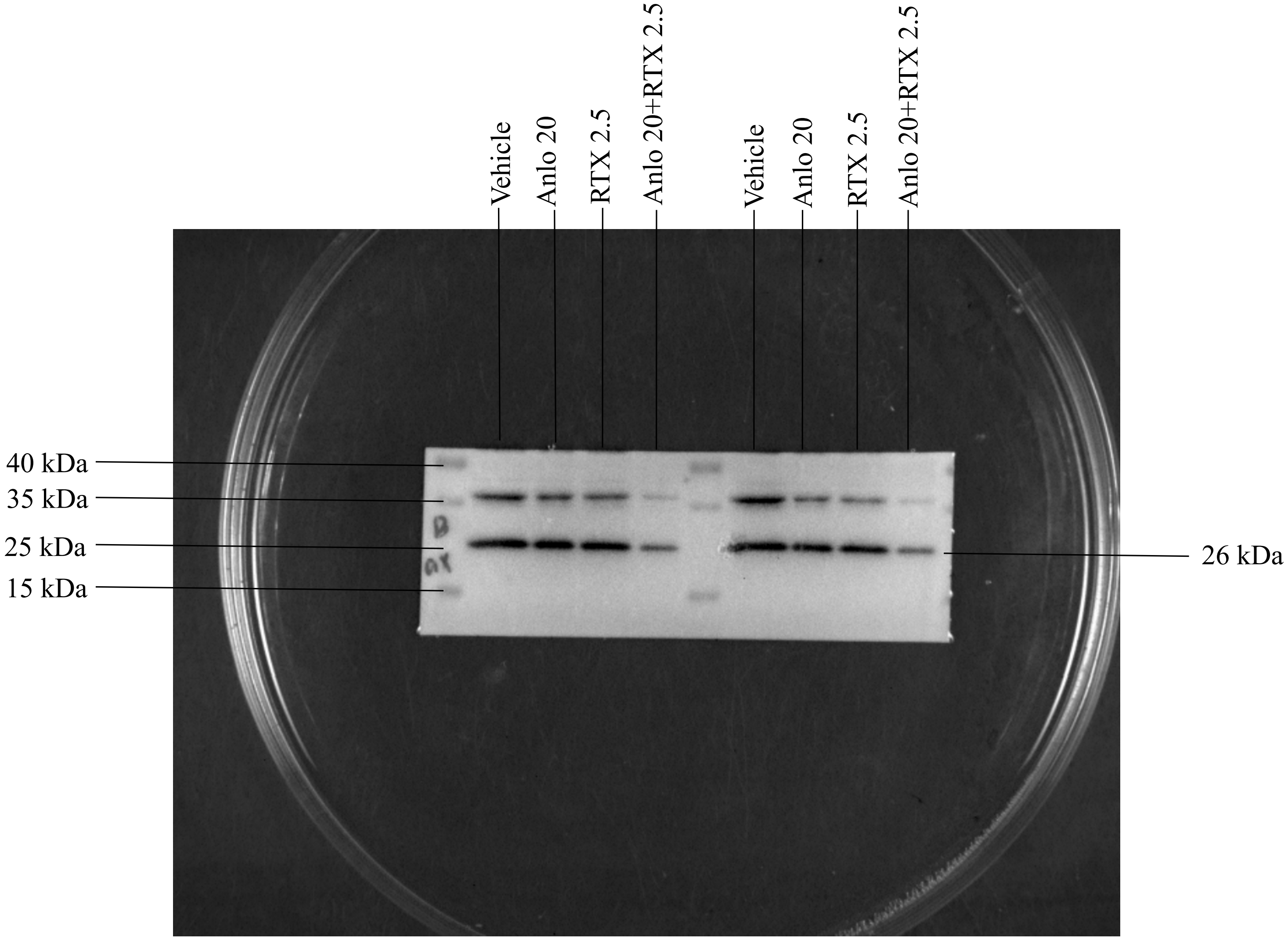

The original Western Blot images of Protein BCL-2 in Figure 7. From left to right: (Left:KYSE-30) : Vehicle, Anlo 20, RTX 2.5, Anlo 20+RTX 2.5; (Right:TE-1): Vehicle, Anlo 20, RTX 2.5, Anlo 20+RTX 2.5. The expected molecular weight is 26 kDa.

MMP9 (KYSE-30)

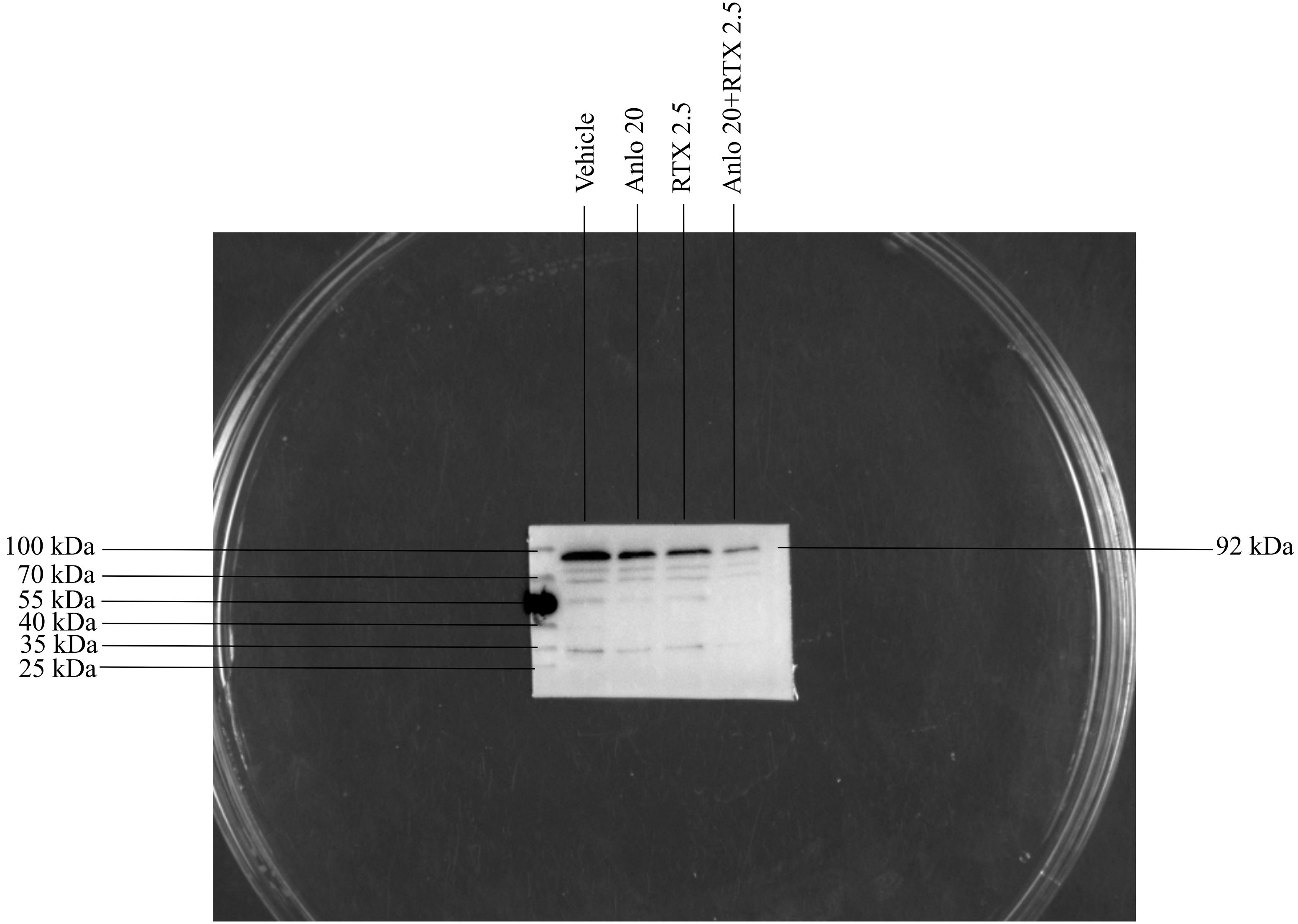

The original Western Blot images of Protein MMP9 in Figure 7 in KYSE-30. From left to right: Vehicle, Anlo 20, RTX 2.5, Anlo 20+RTX 2.5. The expected molecular weight is 92 kDa.

MMP9 (TE-1)

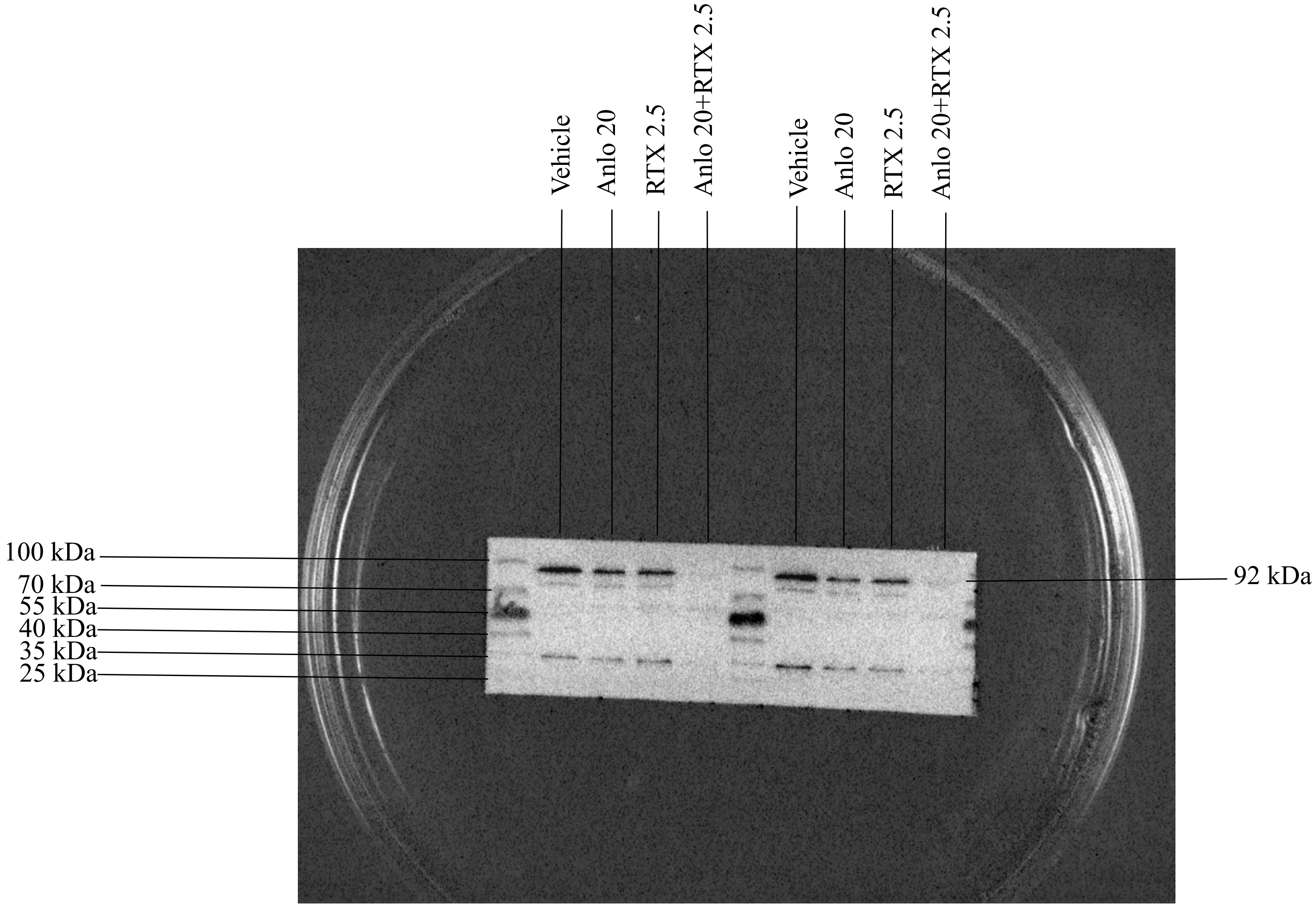

The original Western Blot images of Protein MMP9 in Figure 7 in TE-1. From left to right: Vehicle, Anlo 20, RTX 2.5, Anlo 20+RTX 2.5.

The expected molecular weight is 92 kDa.

P-AKT

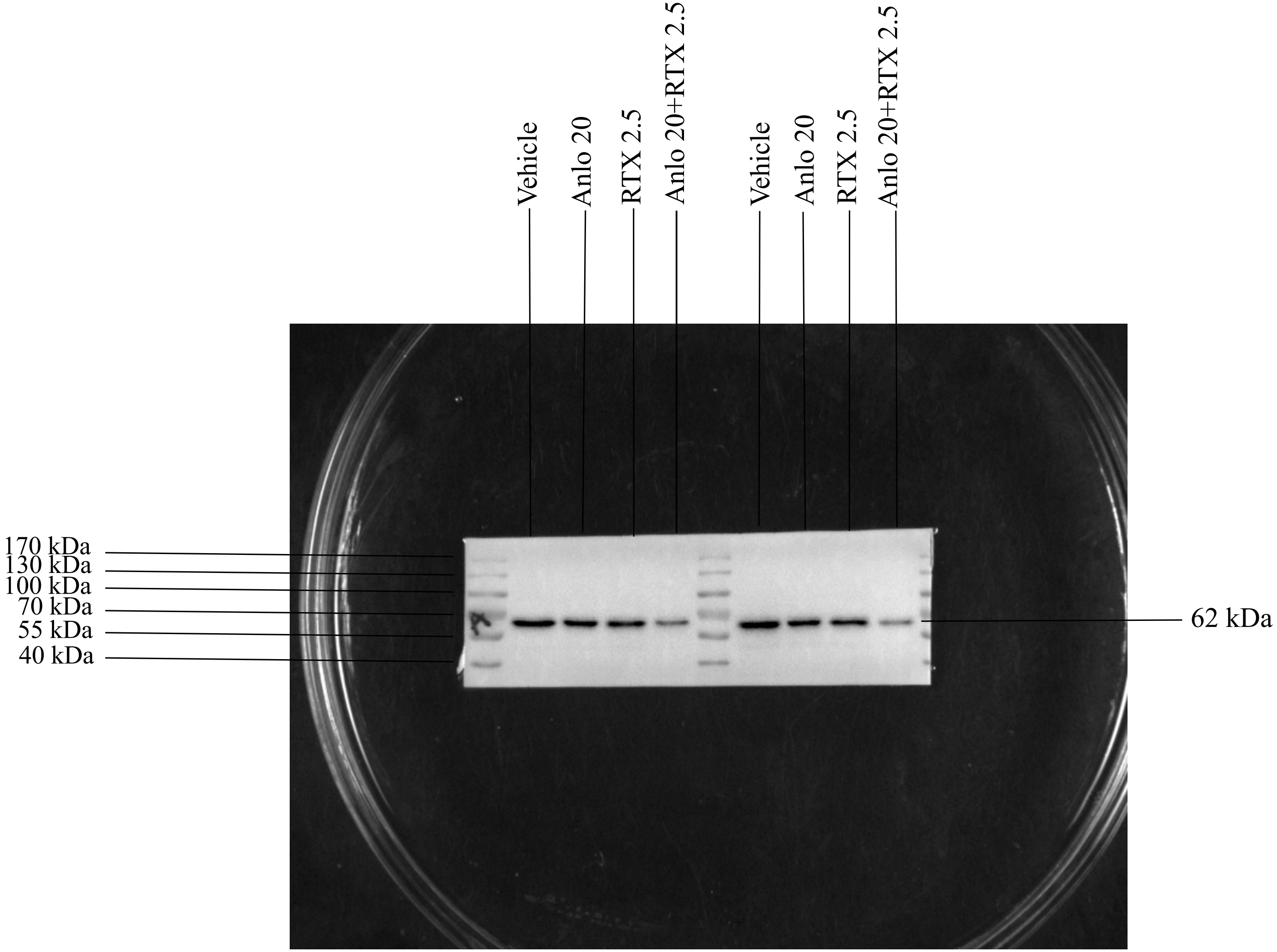

The original Western Blot images of Protein P-AKT in Figure 7. From left to right: (Left:KYSE-30) : Vehicle, Anlo 20, RTX 2.5, Anlo 20+RTX 2.5; (Right:TE-1): Vehicle, Anlo 20, RTX 2.5, Anlo 20+RTX 2.5. The expected molecular weight is 62 kDa.

AKT (KYSE-30)

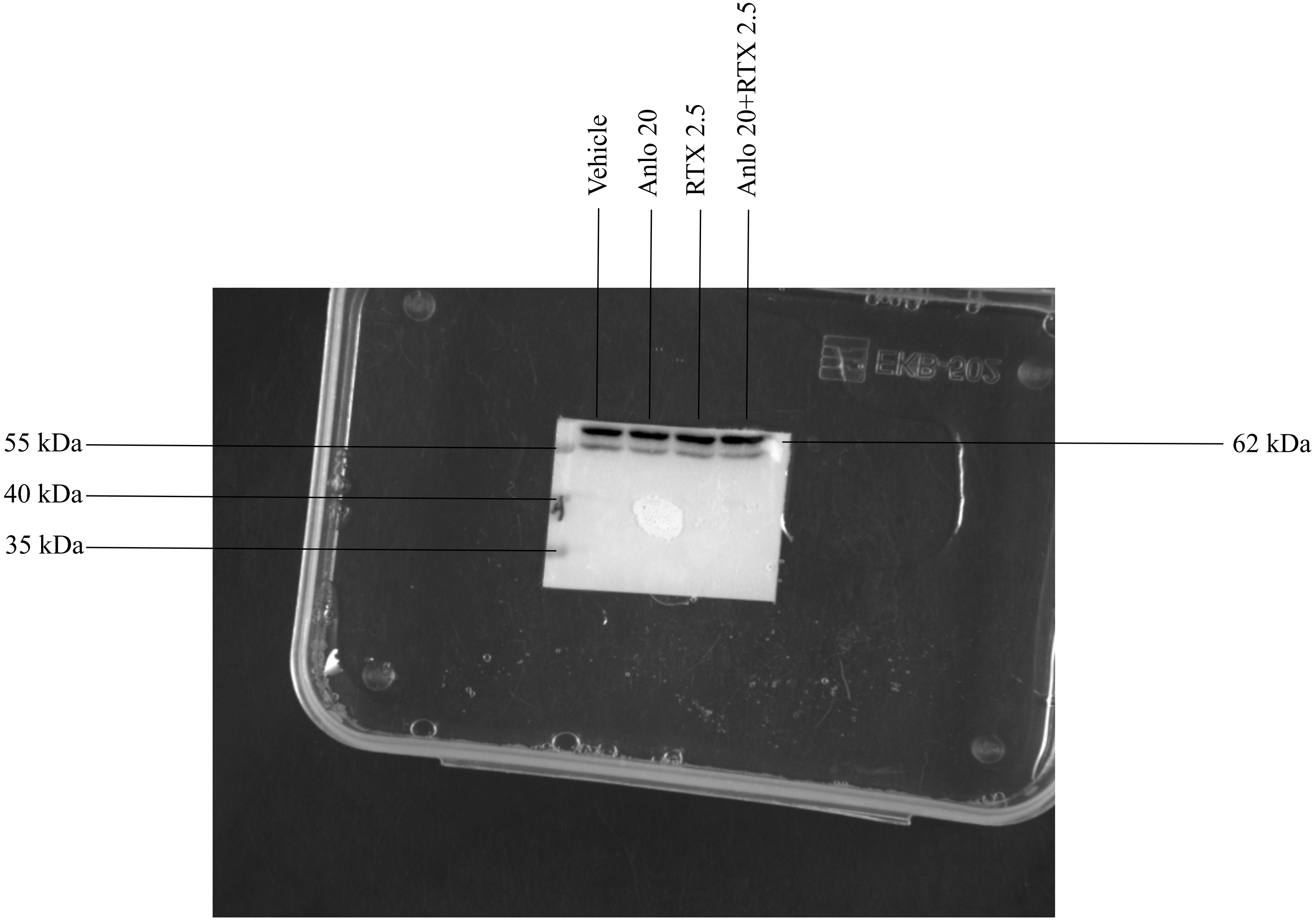

The original Western Blot images of Protein AKT in Figure 7 in KYSE-30. From left to right: Vehicle, Anlo 20, RTX 2.5, Anlo 20+RTX 2.5.

The expected molecular weight is 62 kDa.

AKT (TE-1)

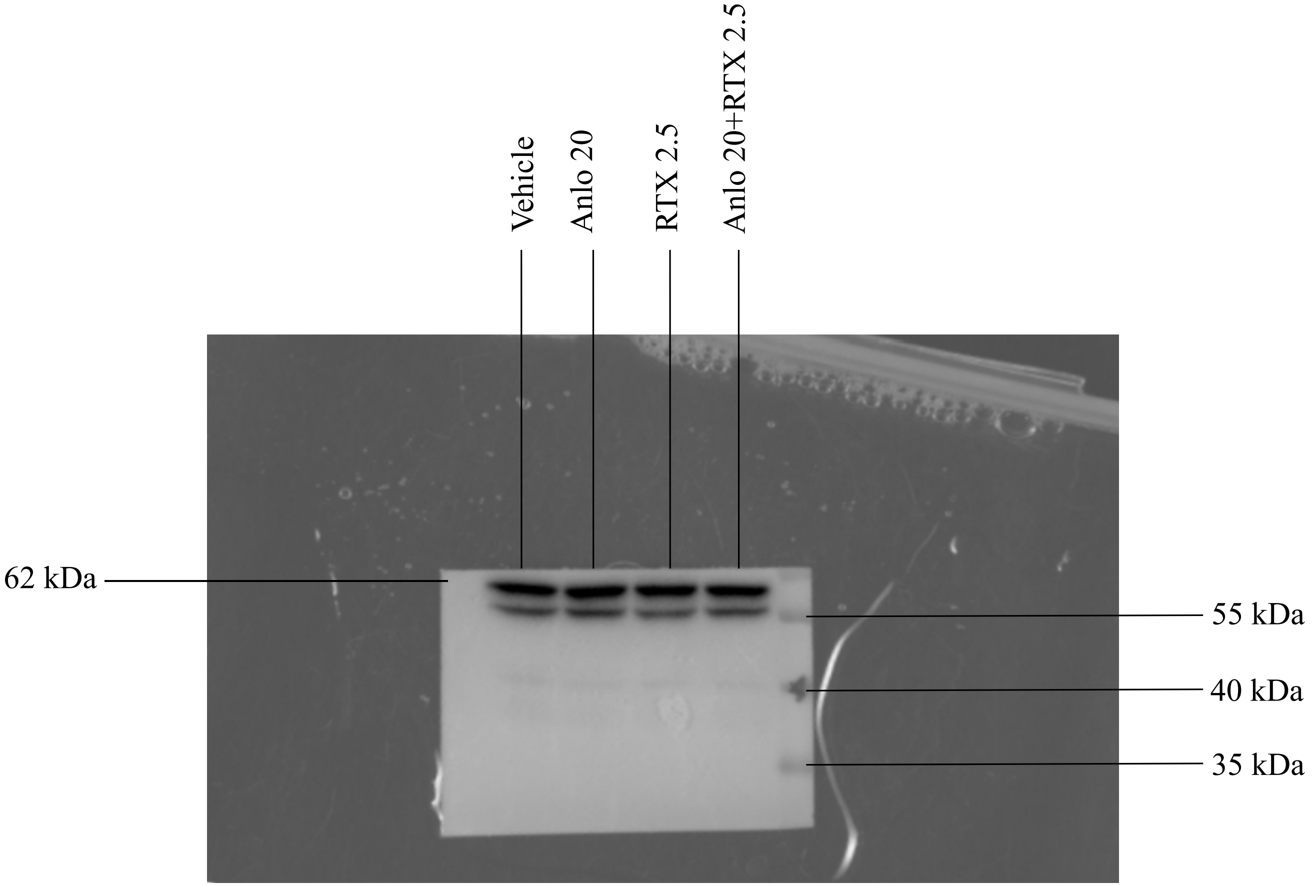

The original Western Blot images of Protein AKT in Figure 7 in TE-1. From left to right: Vehicle, Anlo 20, RTX 2.5, Anlo 20+RTX 2.5. The expected molecular weight is 62 kDa.

P-ERK (KYSE-30)

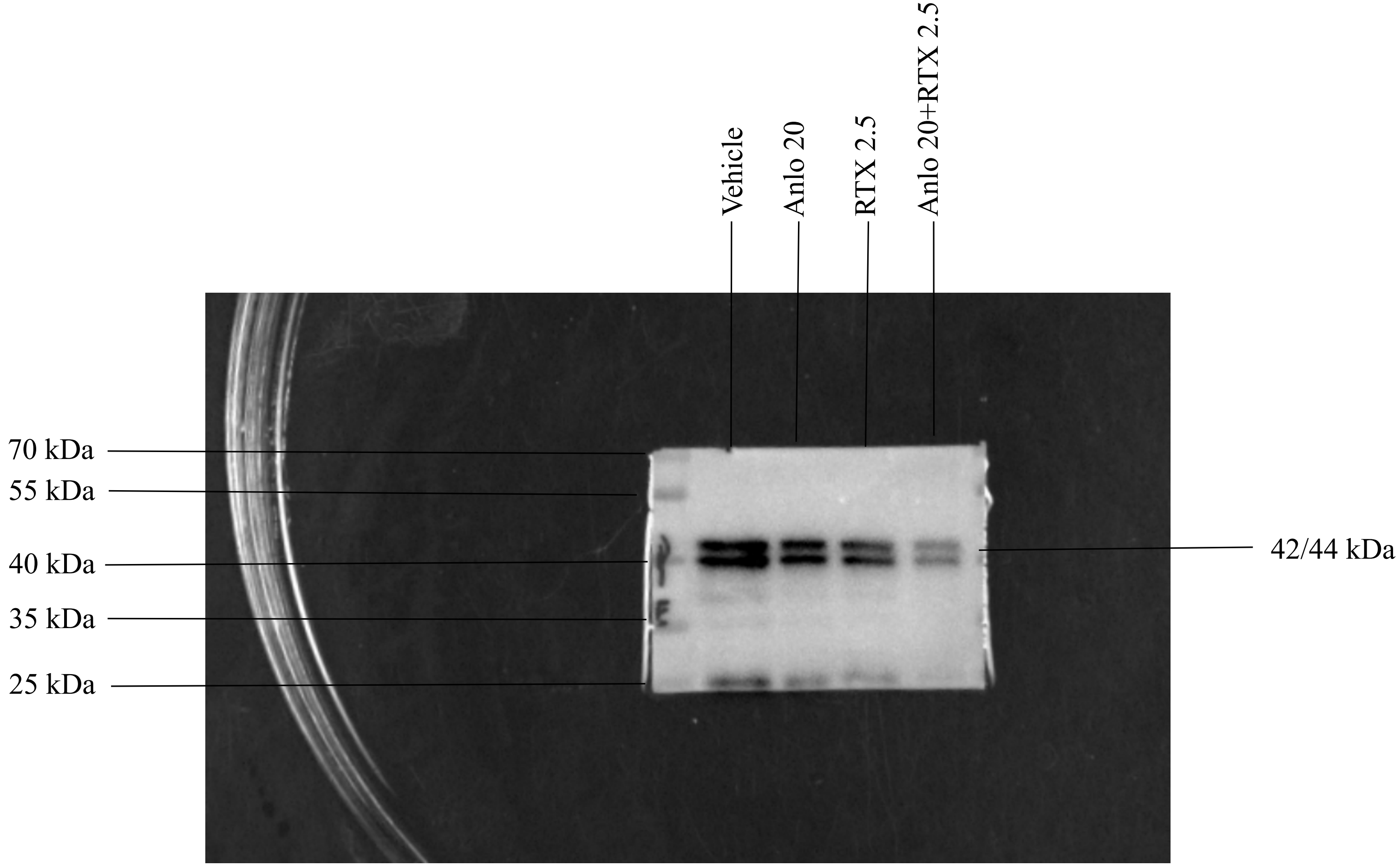

The original Western Blot images of Protein P-ERK in Figure 7 in KYSE-30. From left to right: Vehicle, Anlo 20, RTX 2.5, Anlo 20+RTX 2.5. The expected molecular weight is 42/44 kDa.

P-ERK (TE-1)

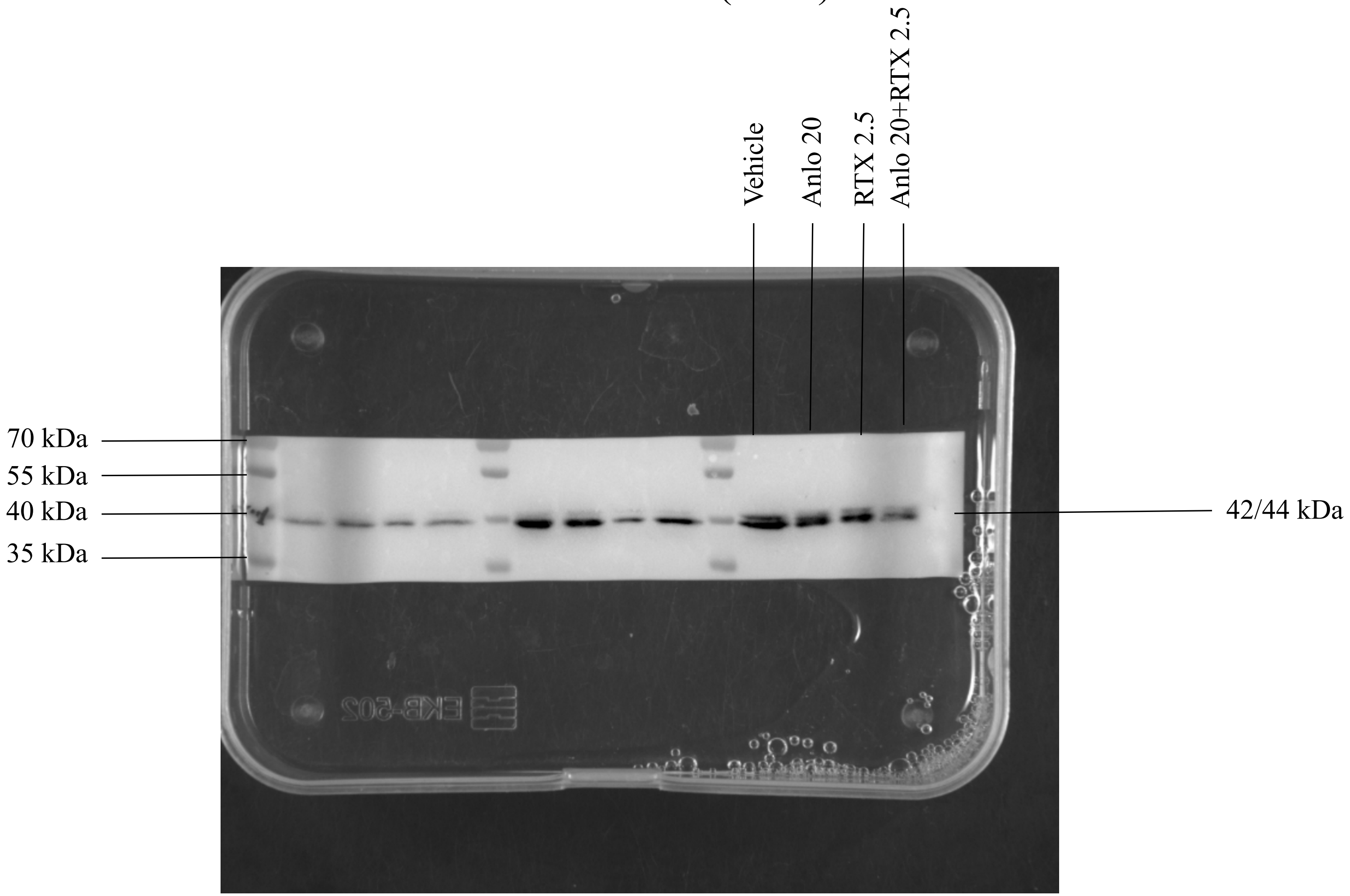

The original Western Blot images of Protein P-ERK in Figure 7 in TE-1. From left to right: Vehicle, Anlo 20, RTX 2.5, Anlo 20+RTX 2.5.

The expected molecular weight is 42/44 kDa.

ERK (KYSE-30)

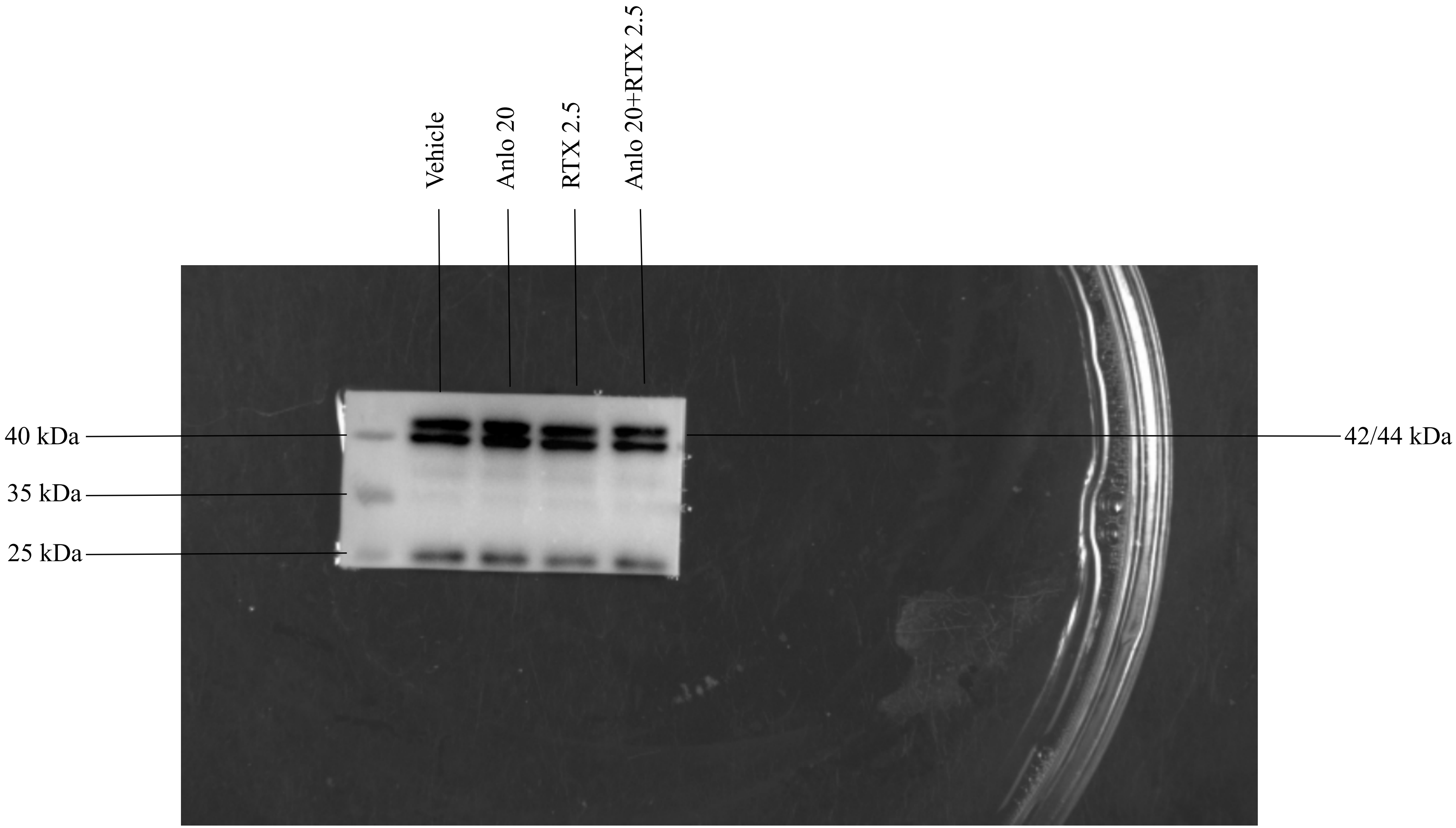

The original Western Blot images of Protein ERK in Figure 7 in KYSE-30. From left to right: Vehicle, Anlo 20, RTX 2.5, Anlo 20+RTX 2.5.

The expected molecular weight is 42/44 kDa.

ERK (TE-1)

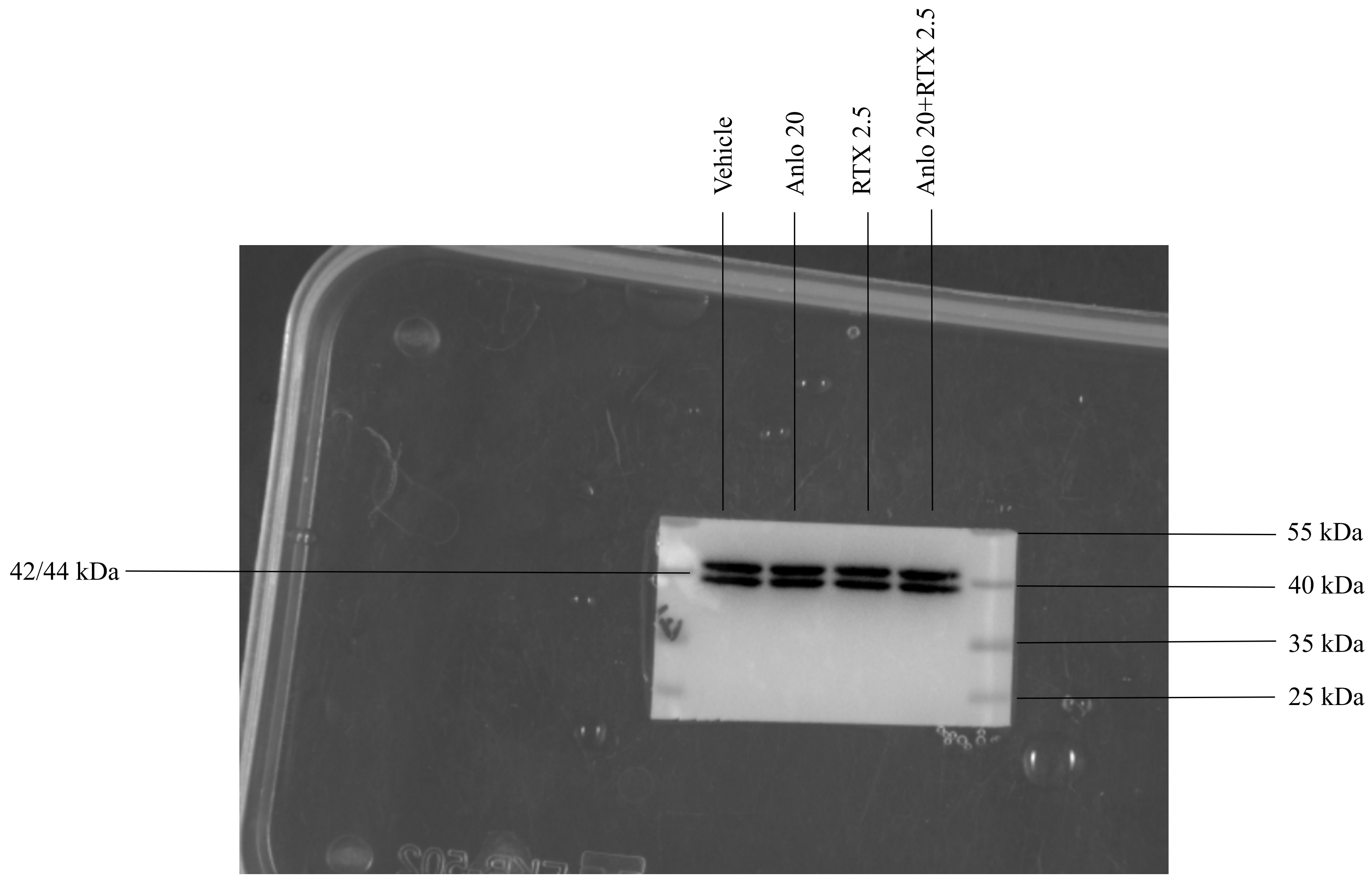

The original Western Blot images of Protein ERK in Figure 7 in TE-1. From left to right: Vehicle, Anlo 20, RTX 2.5, Anlo 20+RTX 2.5. The expected molecular weight is 42/44 kDa.

P-VEGFR2 (KYSE-30)

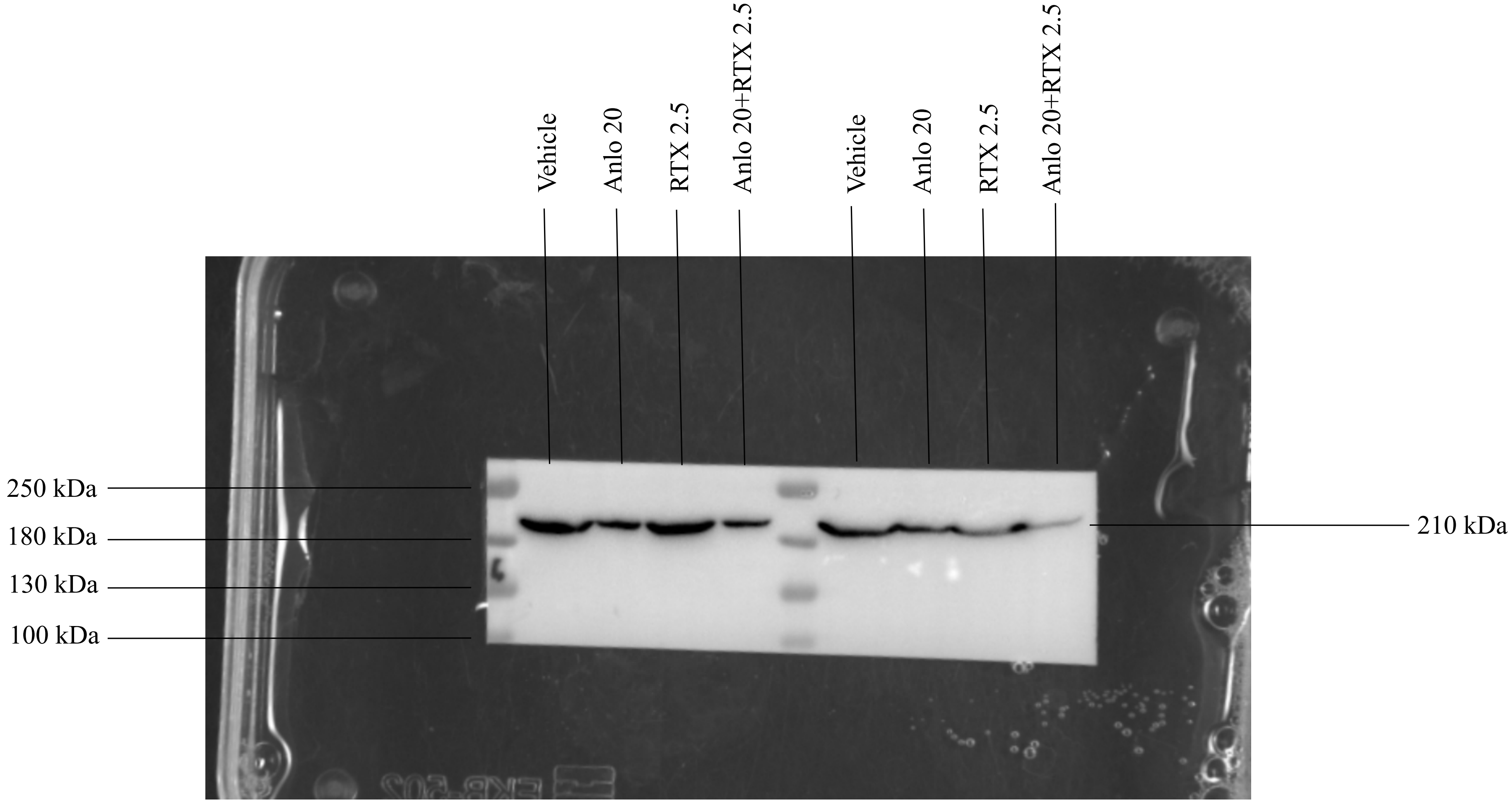

The original Western Blot images of Protein P-VEGFR2 in Figure 7 in KYSE-30. From left to right: Vehicle, Anlo 20, RTX 2.5, Anlo 20+RTX 2.5. The expected molecular weight is 210 kDa.

P-VEGFR2 (TE-1)

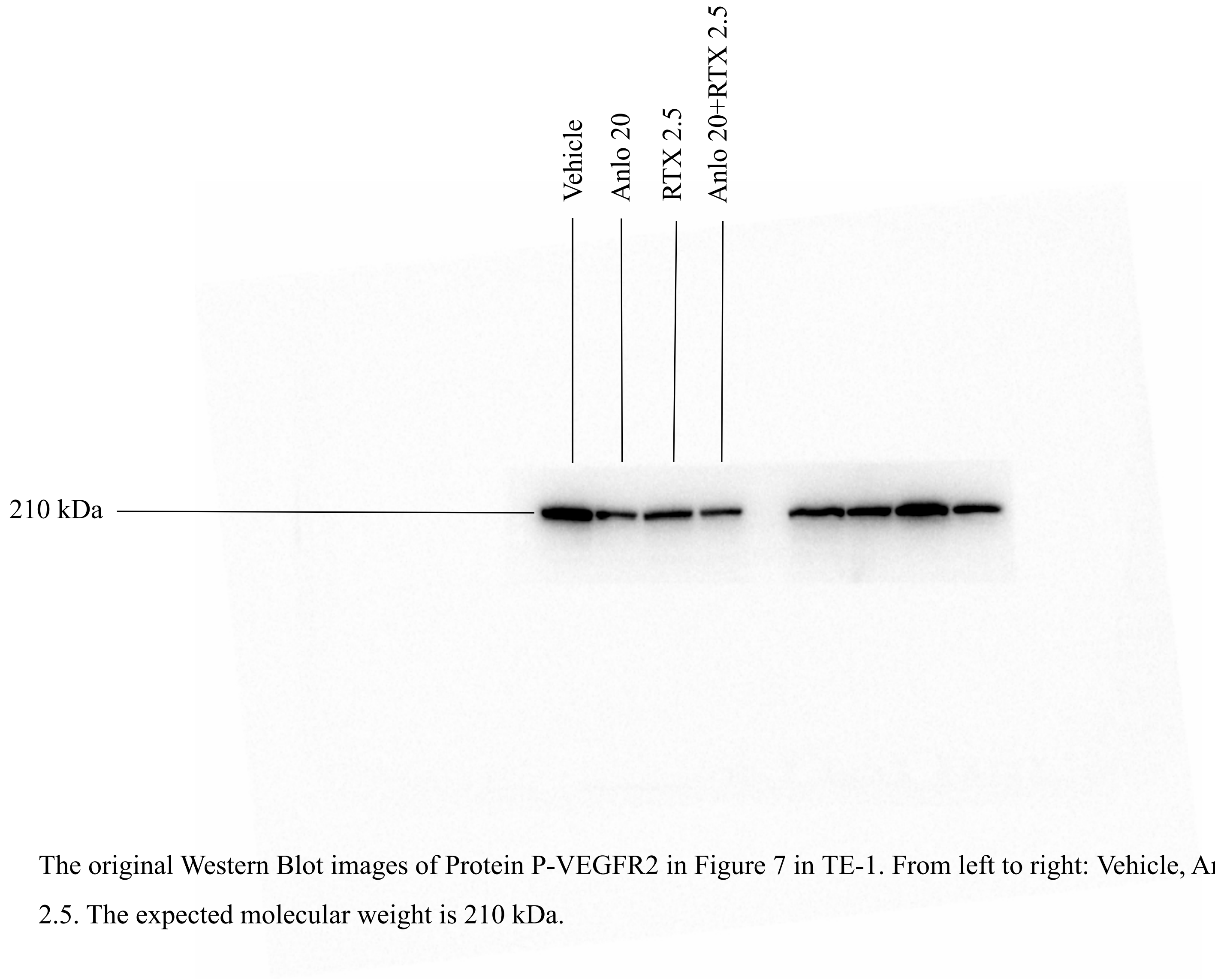

The original Western Blot images of Protein P-VEGFR2 in Figure 7 in TE-1. From left to right: Vehicle, Anlo 20, RTX 2.5, Anlo 20+RTX 2.5. The expected molecular weight is 210 kDa.

VEGFR2 (KYSE-30)

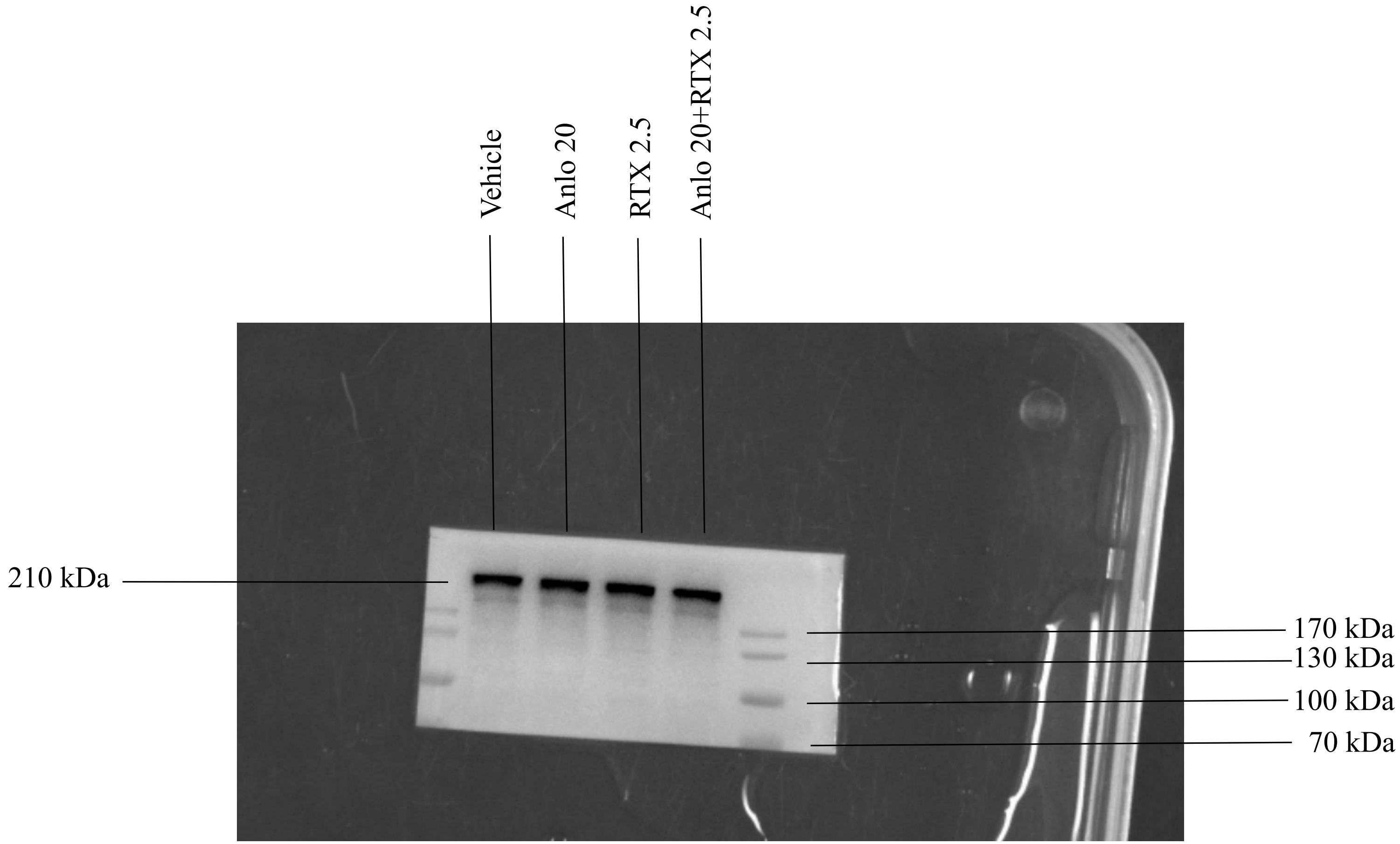

The original Western Blot images of Protein VEGFR2 in Figure 7 in KYSE-30. From left to right: Vehicle, Anlo 20, RTX 2.5, Anlo 20+RTX 2.5. The expected molecular weight is 210 kDa.

VEGFR2 (TE-1)

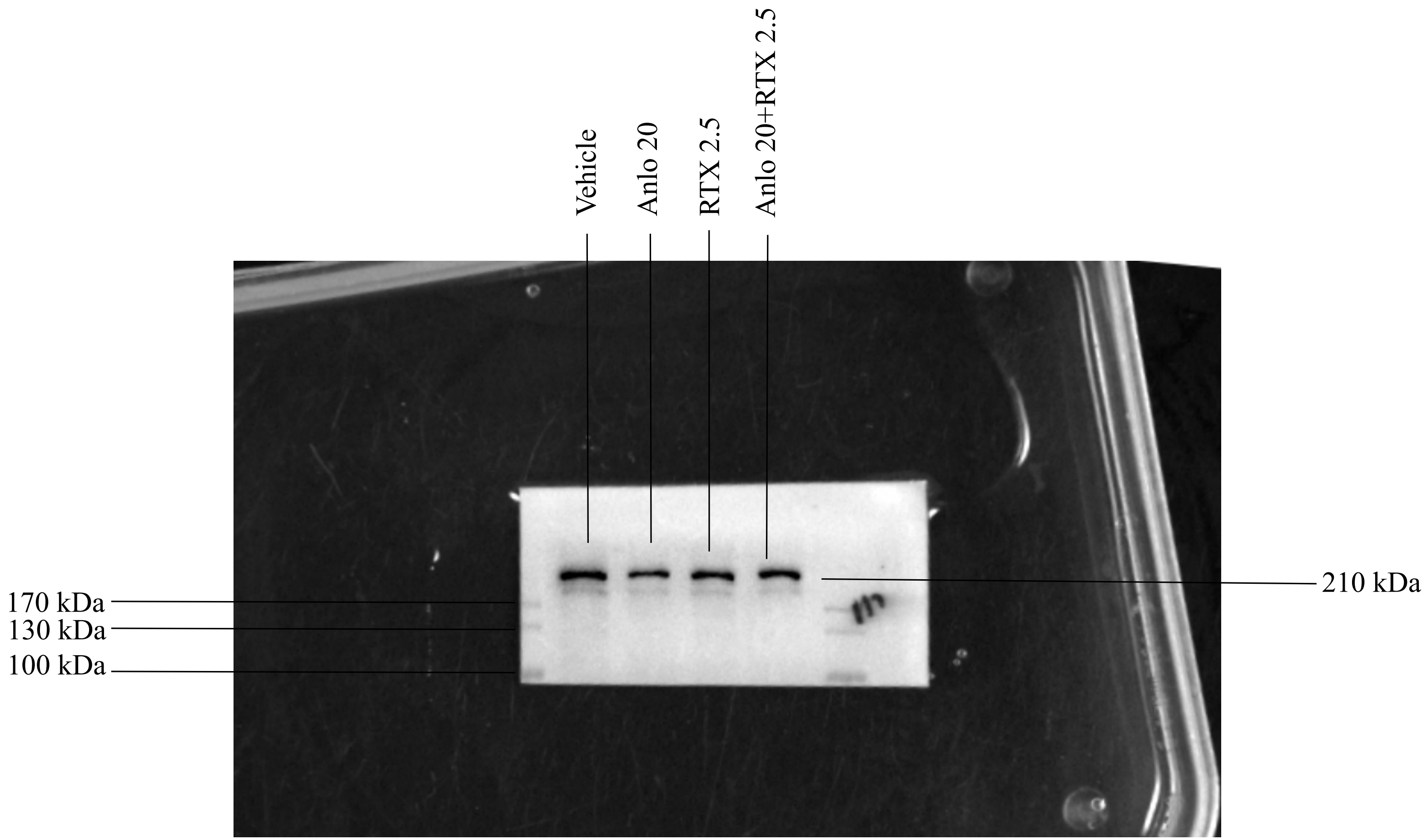

The original Western Blot images of Protein VEGFR2 in Figure 7 in TE-1. From left to right:, Vehicle, Anlo 20, RTX 2.5, Anlo 20+RTX 2.5.

The expected molecular weight is 210 kDa.

Caspase-3

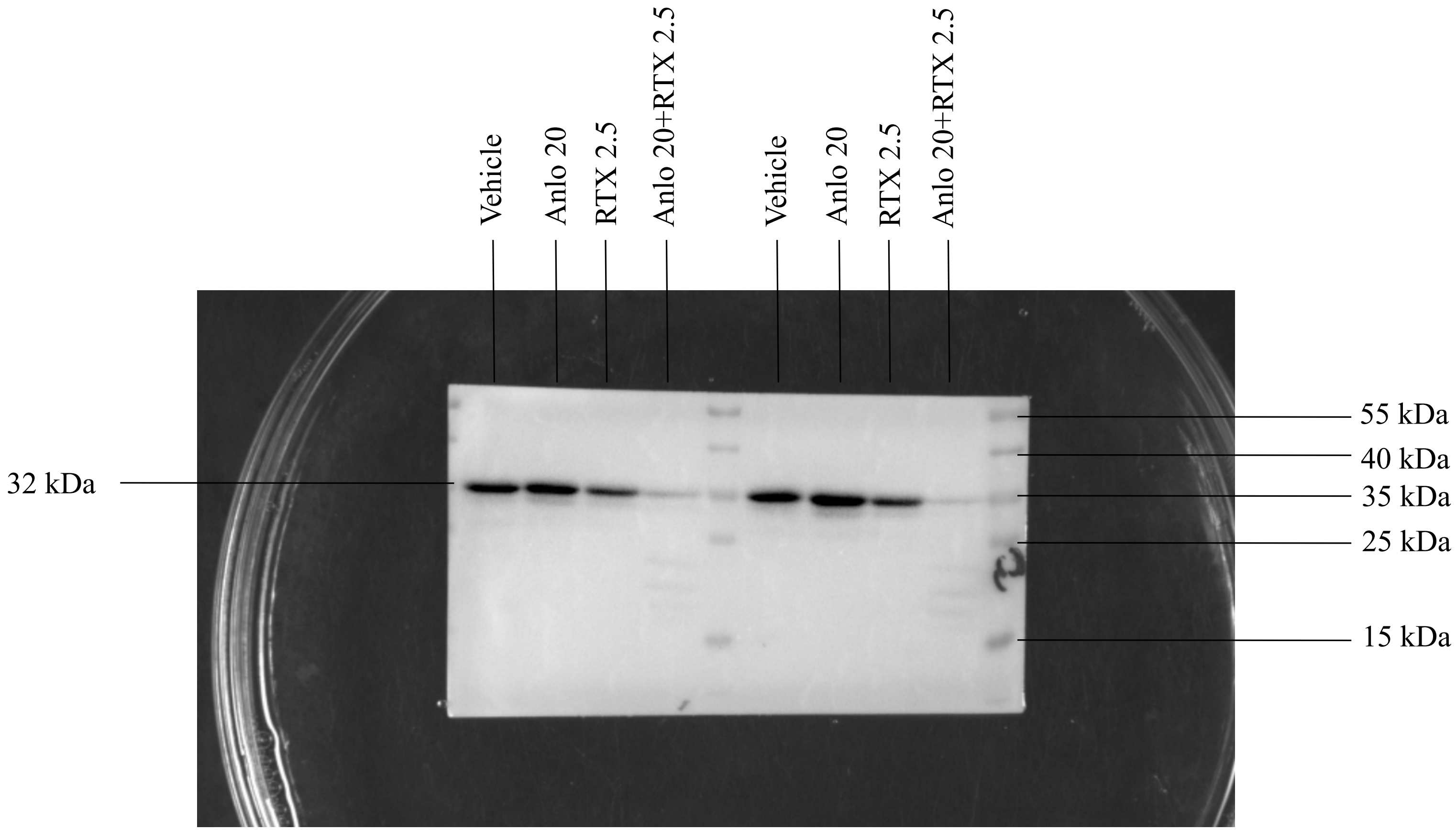

The original Western Blot images of Protein Caspase-3 in Figure 7. From left to right: (Left:TE-1) : Vehicle, Anlo 20, RTX 2.5, Anlo 20+RTX 2.5; (Right:KYSE-30): Vehicle, Anlo 20, RTX 2.5, Anlo 20+RTX 2.5. The expected molecular weight is 32 kDa.

Cleaved Caspase-3 (KYSE-30)

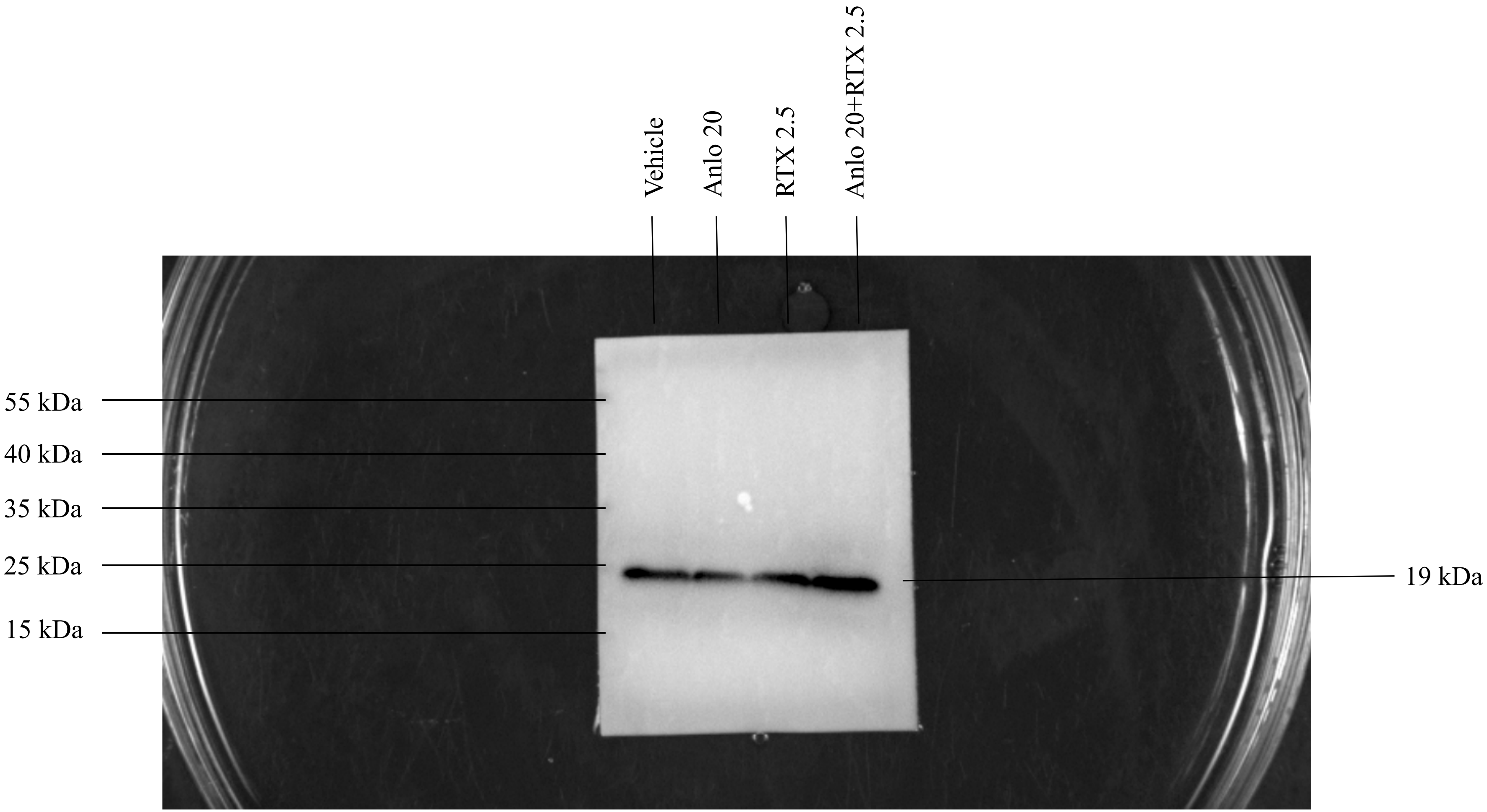

The original Western Blot images of Protein Cleaved Caspase-3 in Figure 7 in KYSE-30. From left to right: Vehicle, Anlo 20, RTX 2.5, Anlo 20+RTX 2.5. The expected molecular weight is 19 kDa.

Cleaved Caspase-3 (TE-1)

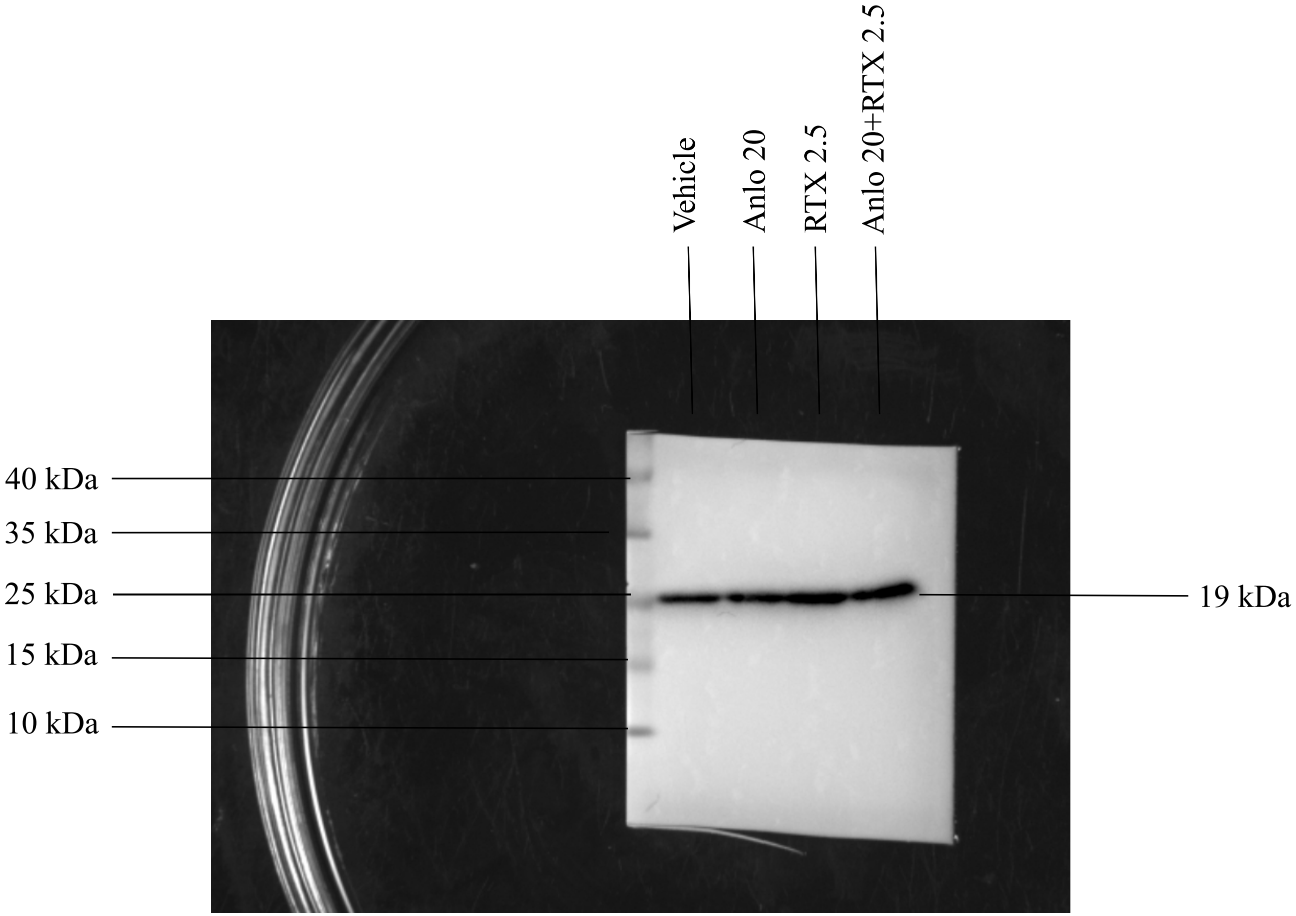

The original Western Blot images of Protein Cleaved Caspase-3 in Figure 7 in TE-1. From left to right: Vehicle, Anlo 20, RTX 2.5, Anlo 20+RTX 2.5. The expected molecular weight is 19 kDa.

ACTIN

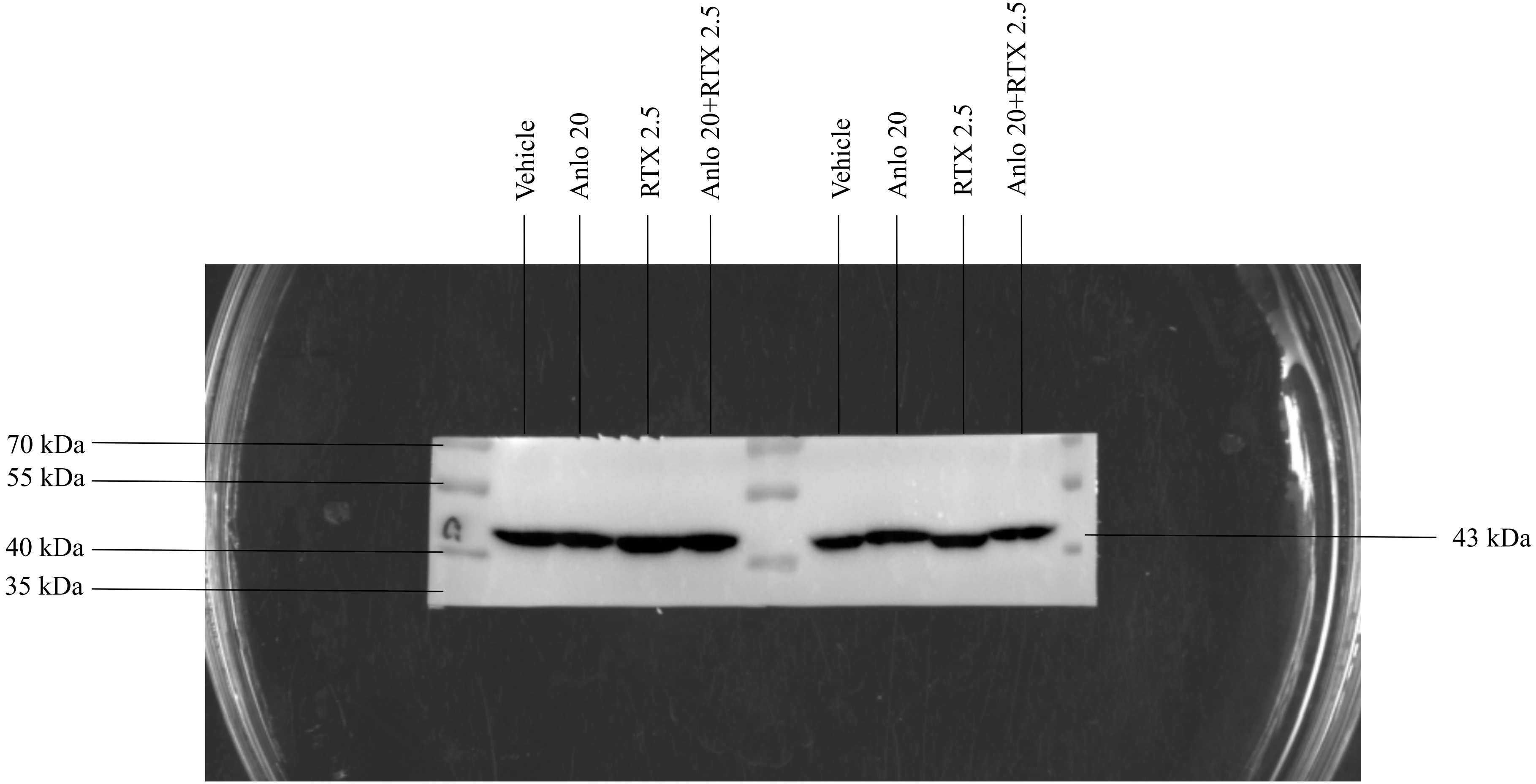

The original Western Blot images of Protein ACTIN in Figure 7. From left to right: (Left:KYSE-30) : Vehicle, Anlo 20, RTX 2.5, Anlo 20+RTX 2.5; (Right:TE-1): Vehicle, Anlo 20, RTX 2.5, Anlo 20+RTX 2.5. The expected molecular weight is 43 kDa.
